# Supplementary figures and images for: Mitonuclear genetic patterns of divergence in the marbled crab, Pachygrapsus marmoratus (Fabricius, 1787) along the Turkish seas
Source: PLoS One. 2022 Apr 5;17(4):e0266506. doi: 10.1371/journal.pone.0266506 (PMC8982882; doi:10.1371/journal.pone.0266506)

Number of alleles vs location sample size

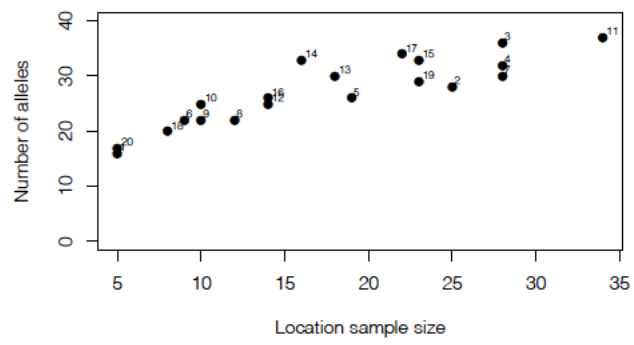

Supplement: S1 Fig — X axis shows the sample sizes of different sampling sites and Y axis shows the total number of alleles corresponding to each site. (PDF) [file pone.0266506.s001.pdf]

K=2

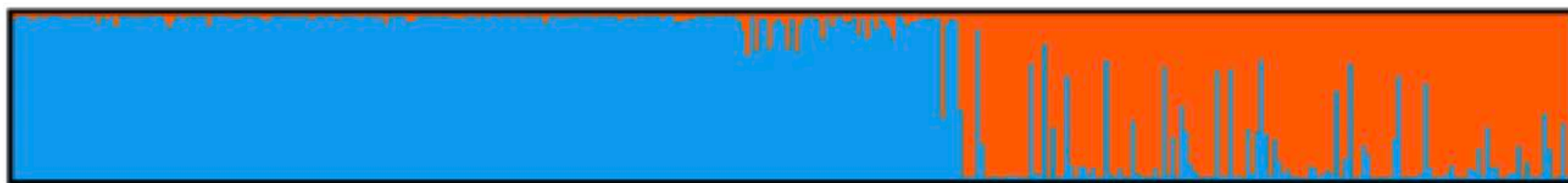

Cluster C: Blue  
Cluster M: Orange

K=3

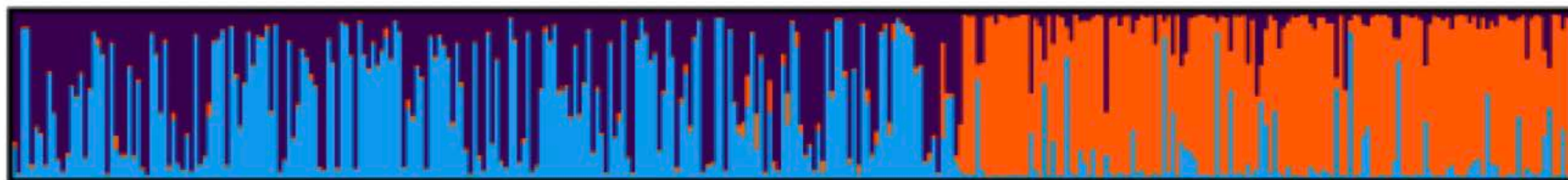

K=4

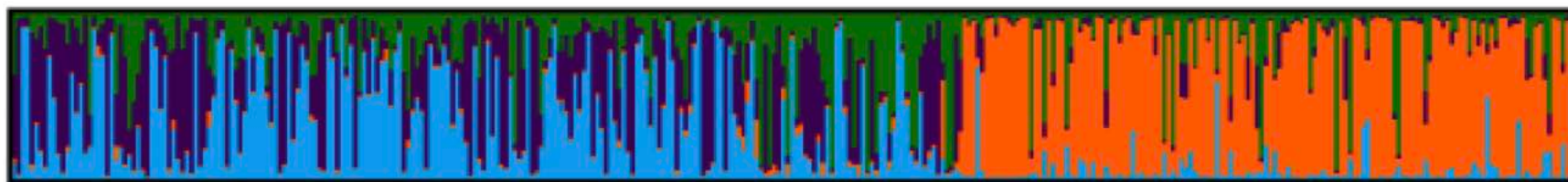

K=5

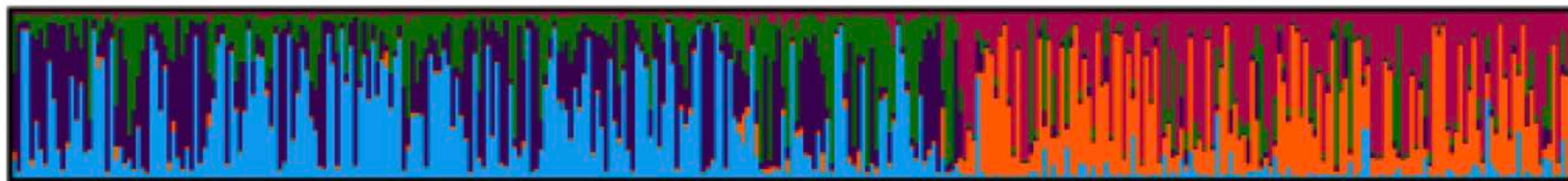

Supplement: S2 Fig — Individuals belonging to cluster C are depicted in blue and those belonging to cluster M are depicted in orange. (PDF) [file pone.0266506.s002.pdf]

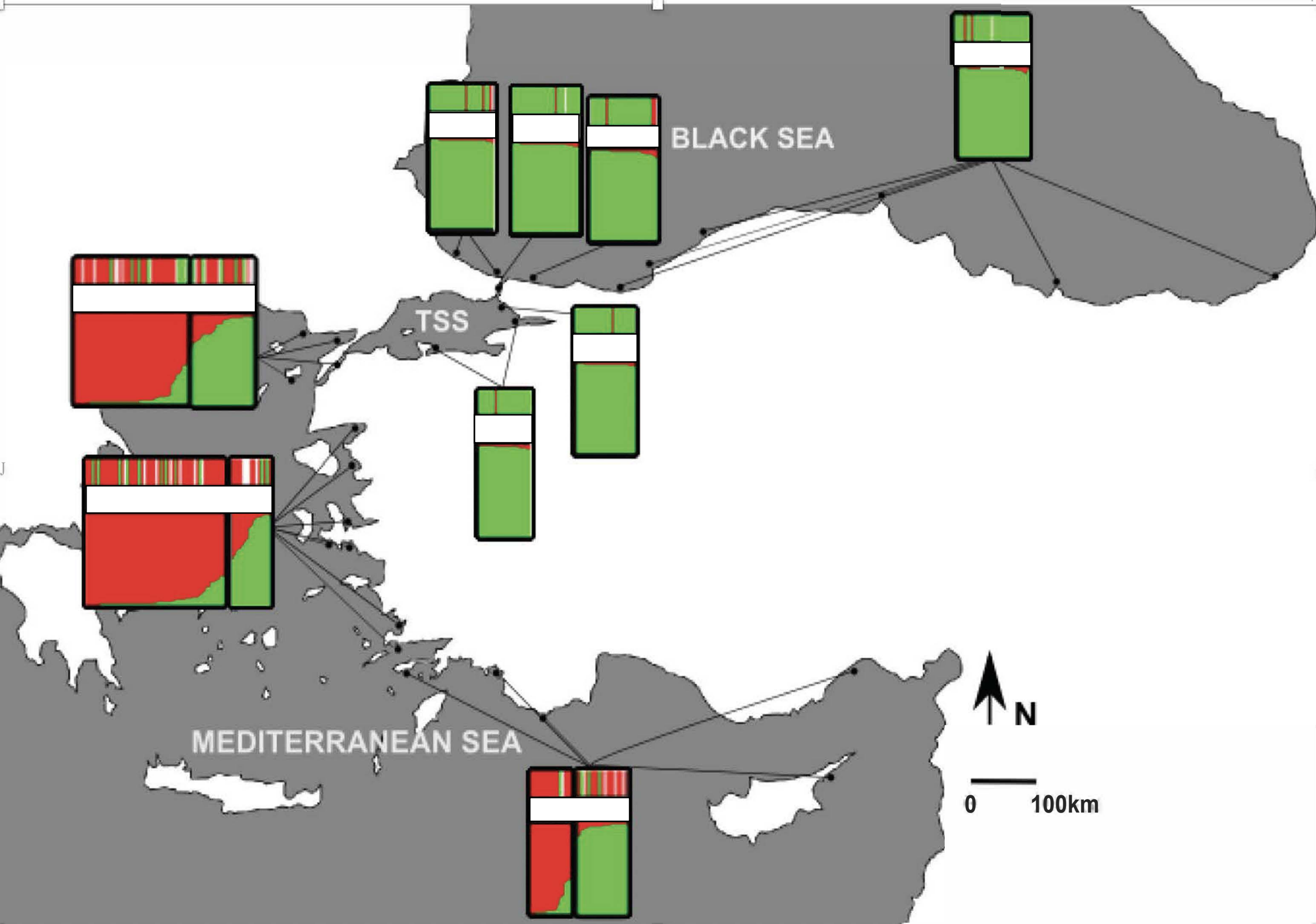

Supplement: S4 Fig — STRUCTURE Q values were ordered in each pooled group of sampling sites (green-population C, red-population M) and are shown in the bottom row. Each bar belongs to one individual. Corresponding mtDNA haplo-groups (green: main haplotype of H7 and its derived haplotypes, red: main haplotypes of H2 and H4 and their derived haplotypes, white: missing data) are indicated on the top row. (PDF) [file pone.0266506.s004.pdf]

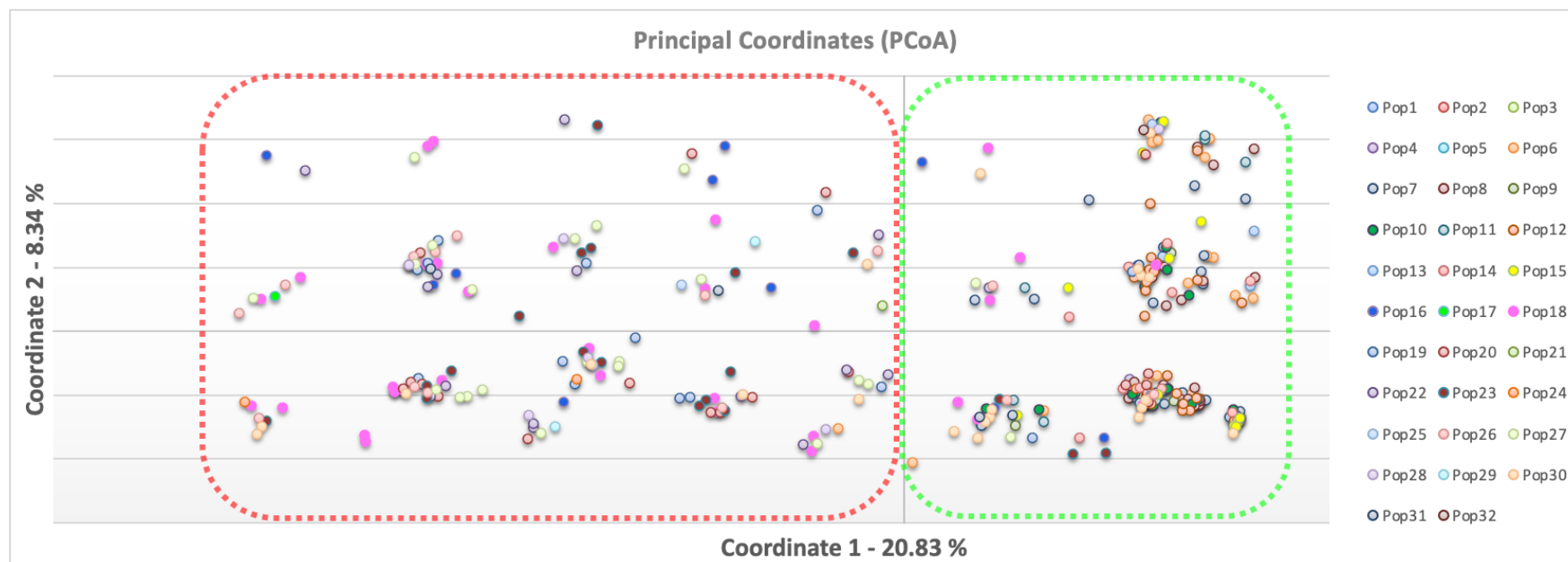

Supplement: S5 Fig — Colors denote original 32 original sampling sites and rectangles denote STRUCTURE clusters (green: C; red: M). (PDF) [file pone.0266506.s005.pdf]

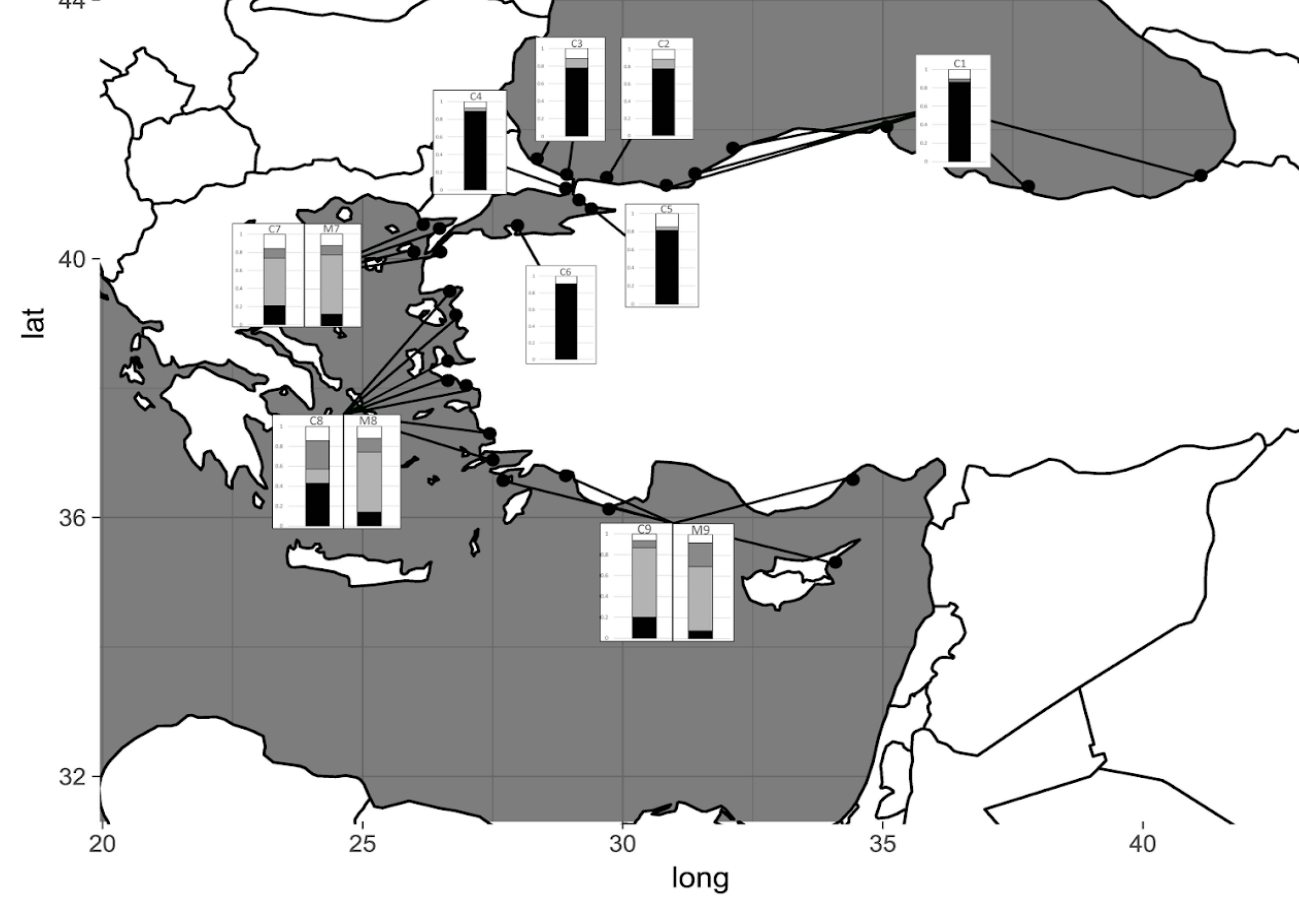

Supplement: S6 Fig — Black: H7; Light gray: H2; Dark gray: H4; White: individuals with less represented haplotypes. (PDF) [file pone.0266506.s006.pdf]
