# Supplementary material for: Mitonuclear genetic patterns of divergence in the marbled crab, Pachygrapsus marmoratus (Fabricius, 1787) along the Turkish seas
Source: PLoS One. 2022 Apr 5;17(4):e0266506. doi: 10.1371/journal.pone.0266506 (PMC8982882; doi:10.1371/journal.pone.0266506)

a) **DeltaK graph:** Optimal K by Evanno is: 2

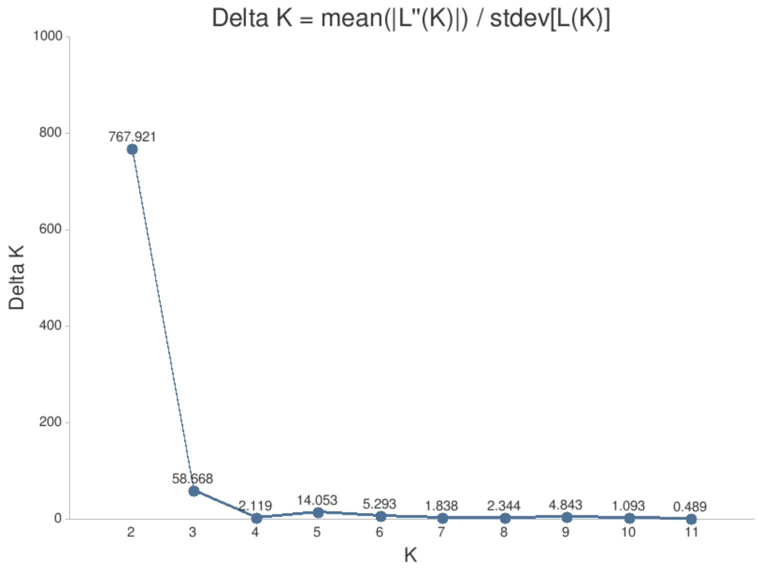

b) **Probability By K graph** Using median values of Ln(Pr Data) the k for which Pr(K=k) is highest: 5

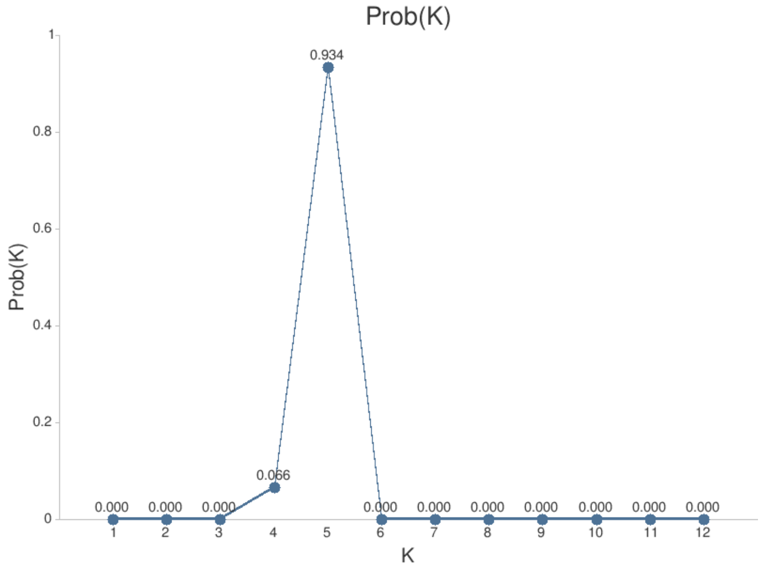

Supplement: S3 Fig — a) Evanno’s statistic, Delta K values as a function of K, K = 1–5, averaged over 20 runs. b) Mean log probability of data (-LnPr) of K = 1–5. (PDF) [file pone.0266506.s003.pdf]
