# Supplementary material for: Mitonuclear genetic patterns of divergence in the marbled crab, Pachygrapsus marmoratus (Fabricius, 1787) along the Turkish seas
Source: PLoS One. 2022 Apr 5;17(4):e0266506. doi: 10.1371/journal.pone.0266506 (PMC8982882; doi:10.1371/journal.pone.0266506)
Supplement: S2 Table — Significant loci determined by HWE test after Bonferroni correction are in bold. (PDF) [file pone.0266506.s008.pdf]

Pop/Loci	pm99	pm101	pm187	pm79	pm108
**1**	20%				
**2**		8%	20%	12%	
**3**					
**4**					
**5**					
**6**					
**7**	11%	**13%**	13%		
**8**					
**9**			24%		
**10**	11%				
**11**	**25%**	9%			**27%**
**12**	17%				24%
**13**	17%		**23%**		28%
**14**	**15%**	16%			
**15**	**25%**	10%			17%
**16**	**22%**				28%
**17**	8%	13%			**21%**
**18**			27%		
**19**	**34%**	12%			**25%**
**20**					
**C**	13%	10%	13%	5%	
**M**	19%	8%	5%		
