# Supplementary material for: Mitonuclear genetic patterns of divergence in the marbled crab, Pachygrapsus marmoratus (Fabricius, 1787) along the Turkish seas
Source: PLoS One. 2022 Apr 5;17(4):e0266506. doi: 10.1371/journal.pone.0266506 (PMC8982882; doi:10.1371/journal.pone.0266506)
Supplement: S3 Table — FST values calculated with FreeNA corrected genotypes and not-corrected genotypes are indicated. (PDF) [file pone.0266506.s009.pdf]

**Locus**	***Fst* not using ENA**	***Fst* using ENA**
pm99	0.0372	0.0308
pm101	0.0141	0.0102
pm108	0.5134	0.5156
pm187	0.0089	0.0187
pm79	0.2178	0.2162
All	0.1186	0.1259
