# Supplementary material for: Mitonuclear genetic patterns of divergence in the marbled crab, Pachygrapsus marmoratus (Fabricius, 1787) along the Turkish seas
Source: PLoS One. 2022 Apr 5;17(4):e0266506. doi: 10.1371/journal.pone.0266506 (PMC8982882; doi:10.1371/journal.pone.0266506)
Supplement: Attachment — Submitted filename: PONE-D-21-13064_reviewer_commented.pdf [file pone.0266506.s012.pdf]

Manuscript
Click here to access/download;Manuscript;CC_ms_20210419_PlosOne.doc

Genetic divergence in microsatellites but not in mtDNA in sympatric populations of the marbled crab, *Pachygrapsus marmoratus* (Fabricius, 1787) along the Turkish seas

Short title: Genetic divergence in microsatellites but not in mtDNA in the marbled crab

Cansu Çetin[1,2,*], Andrzej Furman[1], Evrim Kalkan[3,a], Raşit Bilgin[1,a]

*Corresponding author
email: cansu.cetin@usys.ethz.ch (CÇ)

[1] Institute of Environmental Sciences, Boğaziçi University, Bebek, Istanbul 34342, Turkey.

[2] Institute of Integrative Biology, ETH Zürich, Zürich, Switzerland, Swiss Federal Institute of Aquatic Science and Technology, Dübendorf, Switzerland.

[3] Middle East Technical University, Institute of Marine Sciences, P.O.Box 28, 33731, Erdemli-Mersin, TURKEY

[a]These authors share senior authorship.

Abstract

In this study, we investigated the population genetic structure of the marbled rock crab, *Pachygrapsus marmoratus* along the coasts of Turkey. We genotyped 351 individuals from the Black Sea, the Turkish Straits System (TSS), the Aegean, and the Eastern Mediterranean basins. In order to reveal its evolutionary history and its population connectivity, we used mitochondrial ~~DNA~~ CO1 region and five microsatellite loci. CO1 analyzes also included 610 additional samples from Genbank, which covered most of its distribution range. Microsatellite data analyses revealed two genetically distinct populations of *P. marmoratus* (population C and M). While ~~the range of~~ population C is present in all the sampling locations including the Black Sea and the TSS, population M is restricted to the Turkish Mediterranean coasts including the Aegean and the Levantine basins. In contrast to the divergence in microsatellites, mtDNA data indicated closely related haplotypes, and little genetic divergence. Both microsatellites and mtDNA showed decreased diversity in subpopulations of the TSS and the Black Sea when compared to those along the Aegean and the Levantine coasts. Two possible explanations for the different signals from the two markers, retention of ancestral polymorphisms or gene flow after secondary contact, could not be distinguished from each other.

Introduction

Transition zones are good candidates for studying how molecular markers with different resolution and effective population sizes (1) reflect the evolutionary history of the species. The signature of past ice ages on genetic structure is especially apparent in biogeographical transition zones (2) where expanding post-glacial lineages tend to form hybrid zones for many terrestrial and marine species (3,4). Some of the examples where severe palaeogeographic or palaeoclimatic changes through historical times impacted population genetic structuring or mixing within species in the marine realm are the Almeria-Oran oceanographic front to the east of the Atlantic-Mediterranean interface (5), the Siculo-Tunisian Strait dividing western and eastern Mediterranean (6), North Sea-Baltic Sea transition (7,8) and the Turkish Straits System (TSS) between the Aegean and the Black seas (9,10).

In several marine species, studying biogeographical transition zones with various molecular markers (e.g. mtDNA, microsatellites and restriction-site-associated DNA (RAD) sequencing) helped clarify demographic history of taxa. For example, a secondary contact ca. 11,500 years BP (during glacial retreat) after divergence during Pleistocene glaciations (ca. 270,000 years BP) of the Mediterranean and Atlantic lineages of the European sea bass *(Dicentrarchus labrax)* has been indicated by demographic models (11). Examples of secondary contact over the North Sea-Balthic Sea transition zone include blue mussel *(Mytilus edulis/trossulus) (12)*, Balthic clam *(Limecola balthica) (8)*, vase tunicate *(Ciona intestinalis) (13),* European flounder *(Platichthys flesus)* (14) ~~among many others~~ (reviewed in (15)).

Despite the presence of several studies investigating the role of other transition zones in the Mediterranean in gene flow among locations (5,16,17), studies on the TSS are limited.

The Turkish Straits System (TSS) (Fig 1) is a transition zone between the Black Sea and the Mediterranean Sea by means of two relatively narrow straits (the Bosphorus Strait and the Dardanelles) and an almost entirely landlocked sea (the Sea of Marmara-SoM). The Black Sea has been repeatedly connected and separated from the Mediterranean Sea through the opening and closure of the Dardanelles and Bosphorus Strait during the Quaternary period climatic fluctuations (18–20). These fluctuations have a role in shaping the genetic diversity and diversification in and around the system. In the Black Sea, for example, populations of various taxa have lower genetic diversity in comparison to those in the Eastern Mediterranean, due to founder effects and/or genetic drift in line with their more recent establishment (21–23). The most recent Mediterranean connection was established around 8400 ya, with an inflow of its waters to the Black Sea, and TSS took its current form in terms of its hydrology and current systems around 7500 ya (18,19). The current hydrology of the TSS is characterized by strong stratification (halocline and thermocline) and a two-layer current system, in which brackish water from the Black Sea flows to the Aegean above the denser saline Mediterranean waters that flow towards the Black Sea (24–26).

Fig 1. Sampling locations of *Pachygrapsus marmoratus.*

Those which were previously analyzed on mtDNA (27) and those analyzed in this study (indicated by black triangles and red circles, respectively). Details on sampling sites of this study are indicated in S1 Table.

The two-layered current regime of the TSS might decrease gene flow between populations in the Black Sea and the Aegean. Specifically, it was shown that the lack of surface currents from the Aegean to the Black Sea limits gene flow from the Aegean to the Black Sea for marine species such as anchovy *Engraulis encrasicolus* (9,28), the Mediterranean horse

mackerel *Trachurus mediterraneus* (29), mullets *Liza aurata, L. saliens* and *Mugil cephalus* (30), Atlantic bonito *Sarda sarda* (31,32), one mammal, *Phocoena phocoena* (Linnaeus 1758) (33,34), rock-pool prawn *Palaemon elegans* Rathke 1837 (35,36) , and *Pachygrapsus marmoratus* (27,37). Indeed, studies of (35,38) suggested that the recorded spatial distribution of both genetic types (II and III) within *P. elegans*, across the eastern Mediterranean and Black Sea coasts of Turkey, could be influenced by the impact of the two-layered current regime of the TSS. Additionally, recently established water exchange in the TSS might have created secondary contact zones between previously separated lineages in the Black Sea and the Mediterranean. Despite the above-mentioned studies around the TSS, to reconstruct evolutionary history of species inhabiting this understudied transition zone, there is a need for studies with more detailed sampling, larger sample sizes, and by using markers with higher mutation rates than mtDNA, such as microsatellites or by using more markers (e.g. RAD sequencing).

In this study, we investigated the population genetic structure of the marbled rock crab *Pachygrapsus marmoratus* by using both microsatellites and mtDNA, as a means to reconstruct its evolutionary history in one of the genetically understudied Mediterranean transition zones, the Turkish Straits System. *P. marmoratus* is a good model species that enables investigation of the potential roles played by contemporary and historical factors in shaping the population genetic structure of marine invertebrates in the area. First of all, it is a locally abundant species with a continuous distribution throughout the Mediterranean Sea and the Black Sea. It is also able to tolerate a wide range of salinity (39) and temperature levels (40), which allows it to inhabit different habitats such as the Black Sea (brackish) and the Mediterranean Sea (saline). Both its high fecundity (41) and its extended planktonic larval period (42)high genetic connectivity among n populations. On the other hand, due to its long

planktonic larval duration, the influence of oceanographic fronts can strongly influence its genetic structure (17).

Previous studies have found relatively complex local genetic structure for *Pachygrapsus marmoratus (43–46)*. In terms of mtDNA, there was no separation across Gibraltar Strait and Siculo-Tunisian Strait (44,46), and a weak signature of divergence between Atlantic and the Mediterranean populations (47). The most recent study with the largest sample size (587 individuals, (27) clearly differentiated three geographic groups corresponding to Black Sea, Mediterranean Sea & Canary Islands, and Portuguese Atlantic Ocean (Fig 1). (37)found weak but discernible differentiation in haplotype frequencies between the Black Sea and Mediterranean populations in the mtDNA CO1 region. These results suggest that the CO1 region in this species is more informative in studies with more extensive sampling. On the other hand, several population genetic investigations along microgeographic scales using nuclear microsatellite loci revealed local genetic heterogeneity not related to geography. Patterns of chaotic genetic patchiness (48) have been found across the Italian (43–45), Tunisian (47)), Portuguese (49) coasts and most recently within the Ligurian Sea (50). Based on these insights, the main aims of the present study are 1) to investigate the impact of the Turkish Straits System on genetic connectivity among populations of *Pachygrapsus marmoratus* from the Eastern Mediterranean and Black Sea. 2) to unravel the historical as well as contemporary drivers of population genetic structuring and understand their involvement in shaping spatial distribution of genetic diversity across the TSS biogeographic transition zone using both mitochondrial and nuclear markers.

Methods

Sampling and DNA extraction

A total of 384 specimens of *P. marmoratus* were collected throughout its distribution range along the Eastern Mediterranean and Black Sea coasts of Turkey. Crabs were sampled from 32 locations (Fig 1) between the years 2010 – 2014 at a depth range of 0 – 7 m. The geographic coordinates of the surveyed sampling sites are provided in A1.. After collection, crab samples were preserved in 80-99% ethanol until further processing. DNA was extracted primarily from gill tissues, and occasionally from pereiopod muscles. Gill tissue was preferred because of higher DNA concentration obtained. Genomic DNA was extracted using either Roche High Pure PCR Template Preparation Kit (Indianapolis, USA) or Invitrogen Kit (Carlsbad, CA) following the manufacturers' protocols.

Microsatellite genotyping

384 individuals were genotyped at five microsatellite loci (Pm79 (DQ155410), Pm99 (DQ155418), Pm101 (DQ155416), Pm108 (DQ155414), Pm187 (DQ155419)) using primers designed by (51). Four of the five loci (Pm101, Pm108, Pm187, and Pm99) were amplified with the QIAGEN Multiplex PCR Kit (Hilden, Germany) following the manufacturer's protocols. The fifth locus, Pm79, was amplified based on PCR conditions used by (51), with some modifications. This included 1.2 µl of DNA, 0.5 µl of each primer (10 µM), 2 µl of $MgCl_2$ (2.5mM), 0.4 µl of dNTPs (10 mM), and 0.1 µl of Taq DNA Polymerase (5 U/µl) in a final reaction with a volume of 20 µl. Commercial fragment sizing was done at Cornell University for Pm101, Pm108, Pm187, Pm99, and at Macrogen Holland for Pm79.

Microsatellite fragments were scored manually using Peak Scanner v.1 (Applied Biosystems, USA), and subsequently allele sizes were confirmed using GeneMarker v.2.6.3 (SoftGenetics, USA). Allele sizes were binned using the Excel macro FlexiBin (52). For the subsequent analyses, if a population had less than four individuals, it was merged with the geographically closest population (A1), in accordance with STRUCTURE analysis (see below). This resulted in a total of 20 populations. Genotyping errors due to null alleles, large allele drop-out or scoring of stutter peaks that can potentially lead to deviations from Hardy–Weinberg proportions were analysed using MICRO-CHECKER (53). To estimate null allele frequencies, the equation 1 from (54) was used. The software FreeNA was used (http://www1.montpellier.inra.fr/CBGP/software/FreeNA/) to compute a genotype dataset corrected for null alleles, following the ENA method described in Chapuis & Estoup (2007). Pairwise F_{ST} values calculated with corrected and uncorrected data were compared. Since overall pattern did not change and as there was a slight increase in the F_{ST} values for the loci (Pm79 and Pm108) that contribute the most to the pairwise values in the corrected data, uncorrected data set was used for further analyses to decrease the probability of false positives.

Analysis of genetic diversity and population genetic structure

Individuals with missing data in at least three loci were removed from the data set, resulting in a final sample size of 351 individuals. The STRUCTURE v.2.3.4 (55) analyses comprised several runs with different burn-in lengths and MCMC repeats (50000, 10000, and 20000 for each), with K values ranging from 1 to 12. To infer the number of clusters (K), which fit data the best, the Evanno method (56) was implemented in STRUCTURE HARVESTER (57). Once the best K was inferred, 20 iterations were performed using this value of K. Mean

Q values for these iterations were calculated with CLUMPAK (Cluster Markov Packager Across K, (58), which uses a Markov clustering algorithm that relies on a similarity matrix between replicate runs, as computed by the software CLUMPP (Cluster Matching and Permutation Program) (59). Afterwards, with CLUMPAK, a DISTRUCT (60) graph was produced for the graphical display of the results. In all analyses, uncorrelated allele frequencies were assumed to allow for admixture.

For population analyses, we merged nearby subpopulations if their sample size is smaller than four and if they had similar STRUCTURE assignment probabilities for more statistical power. As a result, 32 sampling points were grouped into 20 (A1). Hardy-Weinberg equilibrium *(HWE)* and linkage disequilibrium *(LD)* were tested in Genepop package in R v.4.7.2 (61,62) via the Markov Chain method (dememorizations = 10000, batches = 1000, iterations = 10000) for the 20 subpopulations and for each pair of loci. The number of alleles (*Na*), the effective number of alleles (*Ne*), the expected heterozygosity (*He*), observed heterozygosity (*Ho*), and allelic richness (A_R) were calculated using the package diveRsity v.1.9.90 of the R software v.3.5.3. In order to check if sample sizes used in this study were representative, the relationship between the number of alleles per subpopulation and sample size was visualized in a graph by using the package PopGenReport (v.3.0.5) of R software (v..3.5.3). An AMOVA (63) was performed using poppr (v.2.8.3) package of R software v. 3.5.3 for the two STRUCTURE clusters and 20 subpopulations. Pairwise F_{ST} and their corresponding significance values (tested with 10000 permutations) were calculated with the software ARLEQUIN v.3.5 (64). Bonferroni correction (65) was used to adjust *P* values for multiple tests, by accounting for the numbers of populations and loci (20x5). The genetic relationships among populations were also evaluated through a principal coordinate analysis

(PCoA) carried out on the distance method of (66), as implemented with the program GenAlEx v.6.5 (67,68).

mtDNA cytochrome oxidase subunit I (CO1) amplification and data analyses

A fragment of the mitochondrial DNA CO1 gene was amplified in 316 individuals of *P. marmoratus* by means of the universal primers (LCO1490 and HC02198), developed by Folmer *et al* (69). 126 of these 316 individuals were sampled and analysed previously by (37). The PCR reaction mix contained 2.5 µl of 10X high fidelity buffer, 2.5 µl of $MgCl_2$ (25 mM), 0.5 µl of dNTPs (10 mM), 0.3 µl of each primer (20 µM), 0.25 units of Taq DNA polymerase (5U/µl), 2 µl of DNA (approximately 50 ng/µl) and 18.45 µl of H_2O for a final reaction volume of 25 µl. PCR was performed according to the conditions described by (69) with some modifications: 10 min at 94°C for initial denaturation followed by 35 cycles of 45s at 94°C for denaturation, 1 min at 50°C for annealing, and 1 min at 72°C for extension, followed by a final extension of 10 min at 72°C.

For the network analysis, 316 sequences from this study were combined with 74 CO1 haplotype sequences from GenBank (corresponding to 610 individuals, with accession numbers JF930650.1-JF930682.1 (44), KX549320.1-KX549335.1 (47), KX529696.1-KX529672.1 (27)), for a total data set of 926 individuals. Sequences were edited manually with Sequencer 5.2.4 (2013 Gene Codes Corporation, Ann Arbor, USA), and aligned with ClustalX (70) The length of sequences used in the final analyses was 490 bp. They were shorter than those of previous studies (e.g. (27) because of the different forward primer used in our study.

A haplotype network was constructed with the software Network v.4.6.1.2 (71), using the median-joining algorithm.

For each of the 20 populations, haplotype diversity (*h*) and nucleotide diversity (*π*) were calculated with DnaSP v5.0 (72). For the pairwise *Φst* computation and AMOVA, ARLEQUIN was used. 15 individuals with STRUCTURE assignment probabilities (Q values) lower than 70% were omitted from the pairwise *Φst* mtDNA analysis.

Deviations from neutral molecular evolution were also tested with ARLEQUIN v.3.5, by calculating Tajima's *D* (73) and Fu's *Fs* (74,75). Significant negative values of these tests may suggest population expansion. R_2 test (76) was applied using DnaSP v.5.0 (72) to detect population expansion, if any. Statistical significance was tested with coalescent simulations based on 5000 simulated resampling replicates.

Results

Analyses with nuclear microsatellite data

Samples from 351 individuals from 32 locations were genotyped at five nuclear microsatellite loci. After Bonferroni corrections, no linkage disequilibrium was observed for any pairs of loci, neither across populations nor in subpopulations. There were relatively high percentages of null alleles in general. The loci Pm99 and Pm108 showed especially high percentages of null alleles in the Mediterranean (between 8-34%) (S2 Table). The locus with the least percentage of null alleles was Pm79, which also had the second highest contribution

to overall F_{ST} values. Genotypic errors due to large-allele drop-out were not observed in any population. After comparing a subset of data with no null alleles with the whole data, we didn't see any differences between population assignment values of individuals in STRUCTURE. Additionally, as mentioned in the methods, F_{ST} values after the ENA correction method of FreeNA software were similar to those uncorrected (S3 Table) ,and thus, we did not correct for null alleles. The relationship between number of alleles and sample size suggested that number alleles tend to stabilize at around 15 individuals (S1 Fig).

STRUCTURE analysis discriminated two clusters. One cluster was widely distributed in all of the sampling locations (referred to as population *C*), and the other was restricted to the Aegean and the Levantine coasts (population *M*). Two clusters (*C* and *M*) coexisted in sympatry across all sampling locations in the Aegean and the Levantine coasts of Turkey (Fig 2). The PCoA also reflected the differences between clusters *C* and *M* (Fig 3), supporting the results of the STRUCTURE analysis. The individuals that comprise the cluster *C* were more similar to each other in terms of their genetic distances, when compared to the cluster *M* (Fig 3). Among the five microsatellite loci, three (Pm108, Pm99, and Pm79) differed the most between the two clusters in terms of their allele frequency distributions (Fig 4). Pm99 and Pm101 were the most polymorphic loci, with 21 and 17 alleles, respectively. Pm79 had three alleles in the Cluster *C* but it was dimorphic in *M* (Table A1). Cluster *C* was fixed for the locus Pm108 except for its individuals in central Aegean (belonging to populations 14 and 15) (S2 Table). Cluster *M* had higher H_e values than cluster *C*, 0.57 versus 0.48, respectively (S1 Table). Individuals belonging to cluster *C* in the Mediterranean (*C1*) had higher H_e values (0.53), when compared to those in the SoM and the Black Sea (*C2*) (0.45) (S2 Table).

Fig 2. STRUCTURE results based on five microsatellite loci at K=2 (on the left) and a map showing the geographical distribution with *P. marmoratus* subpopulations (on the right). Each vertical bar of the STRUCTURE plot represents an individual (y axis) with the colors indicating their associated probability of belonging to one of the two genetic clusters (x axis). Black lines separate the 20 populations. The clusters *M* and *C* are shown in red and green, respectively.

Fig 3. PCoA based on five microsatellite loci for *P. marmoratus* individuals. Colours denote two STRUCTURE clusters: Mediterranean (red square) and Common (green diamonds).

Fig 4. Allele frequency distributions of five microsatellite loci. In each panel, the upper row belongs to the population *M* and the lower row belongs to the population *C*. Numbers in the x-axis correspond to base pairs. The diameter of each circle is proportional to the number of individuals with the corresponding allele.

There was a significant differentiation between sampling sites in the Black Sea & the SoM (1-9) and those in the Dardanelles & the Mediterranean (10-20) (Table 1). It should be noted that Dardanelles samples, although considered as part of the TSS, were differentiated from the other sampling sites in the TSS and the Black Sea but not from the samples in the Mediterranean. Sampling sites in the Black Sea and in the SoM were not differentiated from each other (Table 1). Some geographically close sampling sites in the Mediterranean (such as 19 vs. 17, 13 vs. 15) (Table 1), were differentiated from each other with no clear geographical signal.

Table 1. Pairwise comparisons of nuclear genetic divergence (F_{ST} and Φ_{ST}).

	1	2	3	4	5	6	7	8	9	10	11	12	13	14	15	16	17	18	19	20
1		-0.10	-0.07	-0.07	-0.10	0.00	-0.11	-0.09	-0.08	**0.55**	**0.42**	**0.58**	0.29	**0.38**	**0.75**	**0.51**	**0.45**	**0.74**	**0.67**	0.39
2	-0.04		-0.01	-0.01	-0.01	-0.05	-0.01	-0.01	-0.05	**0.56**	**0.45**	**0.57**	**0.35**	**0.41**	**0.73**	**0.50**	**0.48**	**0.68**	**0.66**	**0.35**
3	-0.02	0.00		-0.02	-0.01	-0.02	0.00	0.00	-0.05	**0.50**	**0.35**	**0.48**	**0.28**	**0.33**	**0.67**	**0.42**	**0.42**	**0.60**	**0.60**	0.23
4	-0.02	0.00	0.00		-0.01	-0.02	-0.01	0.01	-0.05	**0.50**	**0.35**	**0.48**	**0.28**	**0.33**	**0.67**	**0.42**	**0.42**	**0.60**	**0.60**	0.23
5	-0.01	0.01	0.01	0.00		-0.06	-0.02	-0.02	-0.04	**0.53**	**0.37**	**0.50**	**0.30**	**0.36**	**0.68**	**0.44**	**0.44**	**0.61**	**0.61**	0.26
6	-0.03	-0.02	0.01	0.00	0.02		-0.06	-0.04	-0.02	**0.57**	**0.51**	**0.64**	**0.35**	**0.44**	**0.77**	**0.56**	**0.50**	**0.79**	**0.70**	0.52
7	-0.03	0.00	0.00	0.00	0.01	-0.01		-0.02	-0.02	**0.57**	**0.47**	**0.58**	**0.37**	**0.43**	**0.73**	**0.52**	**0.50**	**0.69**	**0.67**	**0.38**
8	-0.01	0.00	-0.01	-0.01	0.01	0.03	0.00		0.01	**0.59**	**0.53**	**0.65**	**0.39**	**0.47**	**0.78**	**0.58**	**0.53**	**0.78**	**0.71**	0.51
9	-0.04	-0.01	0.00	0.01	0.02	-0.03	-0.02	0.01		**0.48**	**0.31**	**0.46**	**0.22**	**0.29**	**0.67**	**0.40**	**0.38**	**0.60**	**0.59**	0.20
10	**0.17**	**0.19**	**0.23**	**0.23**	**0.22**	**0.17**	**0.21**	**0.20**	**0.17**		0.01	-0.02	0.06	0.04	**0.06**	0.00	0.00	-0.06	-0.01	0.03
11	**0.18**	**0.20**	**0.22**	**0.22**	**0.21**	**0.18**	**0.22**	**0.19**	**0.20**	0.00		-0.04	-0.08	-0.08	**0.22**	-0.10	-0.07	0.02	0.08	-0.17
12	**0.15**	**0.19**	**0.21**	**0.21**	**0.20**	**0.18**	**0.20**	**0.18**	**0.18**	-0.02	-0.01		-0.02	-0.06	0.07	-0.02	-0.01	-0.08	0.01	-0.11
13	**0.10**	**0.14**	**0.15**	**0.15**	**0.13**	**0.13**	**0.15**	**0.11**	**0.13**	0.01	0.01	-0.01		-0.06	**0.20**	-0.04	-0.01	0.04	**0.11**	-0.16
14	**0.20**	**0.22**	**0.24**	**0.24**	**0.23**	**0.20**	**0.23**	**0.21**	**0.21**	0.00	-0.01	-0.01	0.00		**0.19**	-0.05	-0.01	0.02	0.09	-0.17
15	**0.20**	**0.24**	**0.25**	**0.25**	**0.24**	**0.23**	**0.25**	**0.22**	**0.22**	0.02	0.02	-0.02	0.03	0.01		**0.20**	**0.16**	-0.02	0.01	0.25
16	**0.22**	**0.24**	**0.26**	**0.27**	**0.25**	**0.22**	**0.25**	**0.23**	**0.23**	0.00	-0.01	-0.01	0.00	-0.03	0.01		-0.06	0.01	0.06	-0.10
17	**0.20**	**0.23**	**0.25**	**0.25**	**0.23**	**0.21**	**0.25**	**0.23**	**0.22**	0.00	0.00	-0.01	0.01	-0.01	-0.01	-0.01		0.00	0.05	-0.06
18	**0.17**	**0.21**	**0.22**	**0.23**	**0.22**	**0.18**	**0.21**	**0.22**	**0.18**	0.02	0.01	-0.02	0.02	-0.01	-0.01	0.01	-0.01		-0.08	0.04
19	0.05	**0.11**	**0.11**	**0.11**	**0.09**	**0.08**	**0.10**	**0.09**	**0.08**	0.05	**0.08**	0.05	0.02	0.07	**0.10**	0.07	**0.09**	**0.09**		0.12
20	0.09	**0.10**	**0.13**	**0.13**	**0.10**	0.06	**0.11**	**0.12**	0.09	0.00	0.03	0.03	-0.02	0.03	0.08	0.05	0.04	0.05	0.01	

The pairwise F_{ST} values (below diagonal) and of mitochondrial genetic divergence (Φ_{ST}) were estimated from sequence divergence data. Significant values are in bold (P threshold <0.0005 for F_{ST} and <0.05 for Φ_{ST}).

Two different AMOVAs were performed by using the microsatellite data set. First, we tested the effect of STRUCTURE and PCoA clusters (*C* and *M*) on the distribution of genetic variation in microsatellites (Table 2). Then, we tested the effect of geography by grouping populations (sampling sites) into three geographic groups corresponding to the Black Sea, the TSS and the Mediterranean Sea (Table 3). In both cases, the largest percentage of variance was explained by variation within populations (66 % vs. 72 %, respectively), followed by variation

between the two clusters (32 %) or three geographic groups (28 %) (Tables 2 and 3). Grouping populations into the two clusters (*C* and *M*) resulted in higher F_{CT} value in comparison to grouping based on geography (F_{CT} value of (0.32 vs. 0.28, respectively) (Tables 2 and 3).

Table 2. Analysis of molecular variance (AMOVA) table on two clusters (C and M) and 20 populations using five microsatellite loci.

Source of variation	SS	Variance components	Variance (%)	F statistics	*P* value
Among clusters	186.82	1.13	31.63	$F_{CT} = 0.32$	**< 0.001**
Among populations/clusters	91.08	0.07	1.92	$F_{PC} = 0.03$	**< 0.001**
Within populations	762.90	2.38	66.44	$F_{SP} = 0.34$	**< 0.001**
Total	1040.80	3.59			

Significant *P* values are in bold (1000 permutations).

Table 3. Analysis of molecular variance (AMOVA) table across three groups according to geography and 20 populations using five microsatellite loci.

Source of variation	SS	Variance components	Variance (%)	F statistics	*P* value
Among groups	205.35	0.91	27.59	$F_{CT} = 0.28$	**< 0.001**
Among populations/groups	72.55	0.02	0.57	$F_{PC} = 0.01$	0.48
Within populations	762.90	2.38	71.84	$F_{SP} = 0.28$	**< 0.001**
Total	1040.80	3.59			

Significant *P* values are in bold (1000 permutations). Three groups are the Black Sea, the TSS, and the Mediterranean.

Main patterns in mtDNA CO1 data

A haplotype network based on 490 bp fragments of the mtDNA CO1 region, included 926 *P. marmoratus* samples from the Mediterranean, the Black Sea, and the Atlantic Ocean (Fig 5). Overall, 80 haplotypes were observed. Since the fragment size in this study was smaller than used in Fratini *et al. (27)*, some of the previously separate haplotypes were grouped together. This decreased the number of 74 haplotypes reported previously in Fratini *et al.* (2011; 2016) to 62. Haplotypes H1-H74 corresponded to haplotypes previously reported in the

studies of Fratini *et al.* (2011; 2016). Among the samples of our study, there were in total 31 haplotypes, 18 of which were recorded for the first time (haplotypes H75-H92). The network showed a star-like pattern with only two bp separating the three most common haplotypes (H2, H4 and H7). The most common haplotype, H2 has a wide geographic distribution covering the entire range of *P. marmoratus*. It is found in 384 individuals (corresponding to 41% of all samples). Around 52% (328 individuals) of the samples from the Mediterranean Sea, 83 % (44 individuals) from the Atlantic Ocean, 6 % (10 individuals) from the Black Sea, and 1 % (2 individuals) from the TSS has this haplotype. The distribution of the second most common haplotype, H7, is geographically restricted to the Black Sea, corresponding to 81% of Black Sea (136 individuals), 87% of the TSS (72 individuals), and 12% of the Mediterranean (73 individuals) samples.

Fig 5. Haplotype network of mtDNA CO1 sequences of *P. marmoratus*.
Network includes samples used in this study and in Kalkan *et al.* (37) together with Genbank sequences corresponding to 926 individuals in total. Each line corresponds to one base-pair difference and the diameter of each circle is proportional to the frequency of that haplotype. Squared names correspond to the haplotypes identified for the first time in this study.

Populations inhabiting the Mediterranean were genetically more diverse than those in the Black Sea and the Sea of Marmara (Table 4). Overall, CO1 diversity was low with 64 polymorphic sites, and with a total of 67 mutations. The average haplotype and nucleotide diversities were 0.72 (SD=0.01) and 0.0027 (SD=0.00006), respectively. Most of the mutations involved synonymous changes to amino acid positions, whereas 10 of the 67 mutations corresponded to non-synonymous sites in the coding region. Nucleotide diversity values in all of our populations were low (< 0.0035); with the ones in the Black Sea and in the SoM having

lower values (0-0.0015) than those in the Mediterranean (0.0014-0.0031). Haplotype diversities ranged from 0.00 to 0.810 (Table 4). The sampling site in the Dardanelles (subpopulation 10) had relatively high nucleotide and haplotype diversities (0.0027 and 0.810, respectively) in comparison to other subpopulations in the TSS (Table 4). The highest nucleotide and haplotype diversities were recorded in the Aegean populations (see sampling sites 10, 13, and 17 in Table 4).

Table 4. Genetic diversity and demographic parameters in *Pachygrapsus marmoratus* populations and subpopulations based on CO1 region.

Pop code	SubPopulation	*N*	*Nh*	*Np*	*h*	*π*	*D*	F_S	R_2
1	Hopa	5	1	0	0.000 (0.00)	0.0000 (0.0000)			
2	Eregli	25	5	5	0.300 (0.12)	0.0010 (0.0004)			
3	Sile	27	5	5	0.393 (0.11)	0.0013 (0.0004)			
4	Kilyos	27	5	5	0.393 (0.11)	0.0013 (0.0004)			
5	Hamsi Limani	19	4	7	0.298 (0.13)	0.0015 (0.0008)			
6	Buyuk Liman	8	1	0	0.000 (0.00)	0.0000 (0.0000)			
7	Caddebostan	25	6	6	0.367 (0.12)	0.0010 (0.0004)			
8	Yalova	12	2	1	0.167 (0.13)	0.0003 (0.0003)			
9	Bandirma	10	2	3	0.200 (0.15)	0.0012 (0.0009)			
10	Canakkale	7	4	3	0.810 (0.13)	0.0027 (0.0005)			
11	Saros	34	8	6	0.633 (0.09)	0.0021 (0.0004)			
12	Enez	10	2	2	0.467 (0.13)	0.0019 (0.0005)			
13	Gokceada	13	5	5	0.744 (0.09)	0.0031 (0.0004)			
14	Dikili	14	4	3	0.654 (0.11)	0.0024 (0.0004)			
15	Urla	20	5	5	0.442 (0.13)	0.0014 (0.0005)			
16	Mugla	13	3	2	0.692 (0.08)	0.0019 (0.0003)			
17	Bodrum	17	6	5	0.765 (0.08)	0.0024 (0.0005)			
18	Palamutbuku	8	3	2	0.464 (0.20)	0.0014 (0.0006)			
19	Kas	19	6	5	0.596 (0.12)	0.0017 (0.0004)			
20	Mersin	4	2	2	0.667 (0.20)	0.0027 (0.0008)			

M	Mediterranean	110	16	14	0.607 (0.05)	0.0020 (0.0002)	**-1.7**	**-12**	0.04
C	Common	206	21	23	0.488 (0.04)	0.0019 (0.04)	**-2.1**	**-19.2**	**0.02**
C1	Common in the north of Dardanelles						**-2.3**	**-18.8**	0.02
C2	Common in Mediterranean						-0.8	-2.7	0.08

Number of individuals (*N*), number of haplotypes (*Nh*), number of polymorphic sites (*Np*), haplotype (*h*) and nucleotide (*π)* diversities per site, Tajima's *D* parameter, Fu's F_s, and R_2 parameter for each population and also for Common population *(C),* its subpopulations in the north of the Dardanelles (*C1*), subpopulations of C in the Mediterranean (*C2*), and Mediterranean (*M*) populations. The significant *P* values are reported in bold.

The same two hypotheses tested with microsatellites (see above section) were also applied to mtDNA. In both cases, largest percentage of variance was explained by variation within populations (52 % vs. 48 %, respectively for grouping based on the geography and on the clusters), followed by variation between the two clusters (36.32 %) or three geographic groups (47 %) (Tables 5 and 6). When we grouped populations based on geography, Φ_{CT} value was recorded as 0.47 (P < 0.001) (Table 5). Secondly, grouping according to microsatellite clusters resulted in a lower Φ_{CT} value than the grouping based on geography (0.39, P < 0.001 vs. 0.47, P < 0.001, respectively) (Table 6). Among the 316 sequenced individuals, only 110 specimens assigned to population M, with a total of 16 haplotypes and 14 polymorphic sites (Table 4). The remaining 206 individuals were found to belong to population C, with a total of 21 haplotypes and 23 polymorphic sites (Table 4). Haplotype diversity of the smaller population, *M,* was higher than the larger one, *C* (0.607 vs. 0.488, respectively).However, the nucleotide diversities of the two populations were comparable (Table 4). The most common haplotypes in population *C* and *M* were haplotypes H7 and H2, respectively (S1 Table). The main genetic differentiation between subpopulations were between those in the Mediterranean region (subpopulations 10-20) and those in the Black Sea and the SoM (subpopulations 1-9),

with Φ_{ST} values ranging from 0.20 to 0.79 (Table 1). The sampling site in the Dardanelles (subpopulation 10) was more similar to sampling sites in the Mediterranean rather than those in the TSS (Table 1).

Table 5. Analysis of molecular variance (AMOVA) table using mtDNA CO1 subunit among three groups according to geography and 20 subpopulations.

Source of variation	SS	Variance components	Variance (%)	F statistics	*P* value
Among groups	68.91	0.34	46.98	$\Phi_{CT} = 0.47$	**< 0.001**
Among populations/groups	8.17	0.01	0.90	$\Phi_{SC} = 0.02$	0.2
Within populations	112.95	0.38	52.12	$\Phi_{ST} = 0.48$	**< 0.001**
Total	190.022	0.73			

Three groups are the Black Sea, the TSS, and the Mediterranean. Significant *P* values are in bold.

Table 6. Analysis of molecular variance (AMOVA) table using mtDNA CO1 subunit among two clusters and 20 populations.

Source of variation	SS	Variance components	Variance (%)	F statistics	*P* value
Among clusters	41.89	0.28	36.32	$\Phi_{CT} = 0.39$	**< 0.001**
Among populations/clusters	44.48	0.12	15.61	$\Phi_{SC} = 0.21$	**< 0.001**
Within populations	104.31	0.37	48.07	$\Phi_{ST} = 0.52$	**< 0.001**
Total	190.69	0.76			

Significant P values are in bold.

In order to infer demographic history of populations, we used three different neutrality tests (the Tajima's *D*, the Fu's F_s , and the R_2 test) (77). Negative values of the Tajima's *D* and the Fu's F_s parameters together with low values of R_2 suggested strong signals for population expansion (75) in the population *M* and in the population *C* inhabiting the Black sea and the TSS (*C1)* (Table 4). Individuals belonging to population *C* in the Mediterranean (*C2*) did not show any significant signals of population expansion (Table 4).

Discussion

Our results show that different markers, microsatellites and mtDNA, show much lower genetic diversity in the Black Sea and the SoM in comparison to the Mediterranean Sea. Most probably, stronger genetic drift in the Black Sea and the SoM as a result of their isolation in the Pleistocene climatic fluctuations is the main reason for this pattern (18–20). The differentiation between different basins (the Black Sea, the TSS, and the Mediterranean) is very strong in mtDNA. This is not very surprising given that effective population size (N_e) of mtDNA is one-quarter of that nuclear genome; thus it will sort faster when there is a genetic break between lineages (78). On the other hand, the distribution of genetic variation in microsatellites shows the signature of more contemporary gene flow between the Black Sea and the Mediterranean populations.

One of the assumptions of this study was that Dardanelles is part of the TSS. However, the genetic differentiation between the *P. marmoratus* samples from Dardanelles and those from the TSS and the Black Sea, in both mtDNA and microsatellites, suggests that different dynamics influencing gene flow might be in effect. Complex oceanographic characteristics of Dardanelles which entails a two-layer exchange driven by density difference and sea-elevation difference between the basins of Aegean and the SoM might be a mechanism explaining this pattern (79,80). For example, it was shown that the high salinity inflow from the Aegean does not completely reach the SoM, around 43-45 % returning back to the Aegean by mixing into the upper layer before reaching the Nara Pass in the Dardanelles (79–81). Since the location of *P. marmoratus* samples from Dardanelles is on the Aegean side of the Nara Pass, its larvae might be reaching there by following the upper layer of the current from the Aegean.

Additionally, in terms of temperature and salinity, Dardanelles is more similar to Aegean Sea rather than the SoM (82). Thus, adaptive processes might also have a role in explaining similarity of Dardanelles samples to Aegean samples. A similar pattern has also appeared in *P. elegans* (Kalkan *et al.* 2021, submitted), so that Dardanelles samples (N=4) were more similar to Aegean rather than TSS in their mtDNA CO1 haplotypes. More detailed sampling with more sample size around the Dardanelles is needed to investigate this pattern and to see if complex oceanographic currents reflect connectivity of different sites in this transition zone.

Our analyses with microsatellites reveal two distinct genetic populations in *P. marmoratus* (population *C* and *M*) along Turkish coasts. In contrast to divergence in microsatellites, mtDNA data indicated closely related haplotypes, and did not reflect the same degree of divergence in the Mediterranean. This difference can be due to either recent gene flow between these two subpopulations or ancestral polymorphisms that did not yet get sorted (78). In each of the two scenarios presented below, the hypothetical origins of these two populations are discussed by referring to the geological history of the regions of interest. However, it should be noted that, since the detailed biogeographic and evolutionary history of *P. marmoratus* has not yet been constructed throughout its distribution range, proposed dates of origins of these two populations is still disputable.

Population C: An ancestral population along Turkish coasts

According to this scenario, *C* was a local population around the eastern Mediterranean before the last opening of the Dardanelles, about 9,000 years BP (20). The ancestral population *M*, on the other hand, might have originated in the western Mediterranean or in the northeastern Atlantic, but had not reached eastern Mediterranean before the Last Würm Glaciation (24,000-10,000 ya). When the Mediterranean Sea levels exceeded that of the Black Sea around 9,200-

7,500 years BP, Mediterranean water began to flow towards the Black Sea (18,19). In about 1,000-1,500 years, the salinity of the Black Sea became high enough to support various Mediterranean species (20). Thus, *P. marmoratus*, with its pelagic larvae, could have populated the Black Sea during that period. During this range expansion, the expanding population was likely to have undergone a population bottleneck, leading to the loss of genetic variation in the Black Sea. The lower average He values of population *C* (0.48) compared to population *M* (0.57) support this idea. Population *C* had lower haplotype diversity (0.49) of CO1 region than population *M* (0.60). These values are lower than the average haplotype diversity (*h*=0.81) found by (47), suggesting the role of genetic drift. Especially, subpopulations of *C* in the Black Sea and the SoM (1-9) had the lowest haplotype diversity values (ranging from 0-0.4). Low diversity in Black Sea subpopulations with respect to those in the Mediterranean in terms of mtDNA has also been shown in other marine species, including anchovy (9) and a chaetognath (83). Genetic drift in the Black Sea and the SoM populations is also supported by higher *He* values of population *C* in the Mediterranean (between 0.49-0.54), when compared to in the Black Sea and the SoM populations (between 0.42-0.45) in microsatellites. On the other hand, higher *He* of population *C* in the Mediterranean could also be due to local gene flow from the sympatric population *M* to sympatric subpopulations of *C* in the region.

Subsequent to range expansion of population *C* to the Black Sea, a more recent range expansion might have resulted in population *M* inhabiting the Mediterranean coasts of Turkey. Around 7,000 years BP, the current pattern in the TSS formed, and the Black Sea water began to flow towards the Mediterranean (18,19,84). Thus, if population *M* reached eastern Mediterranean after 7,000 years BP, the current pattern may have impeded the incoming population *M* from inhabiting the Black Sea and the SoM. Signs of population expansion for *M* in the Eastern Mediterranean, but not for the *C* subpopulations in the same region support this scenario. As suggested in this scenario, if *M* was the population dispersing into the Turkish

Mediterranean coasts, unidirectional gene flow of local population *C*'s mtDNA into that of *M* could be an explanation, creating similarity in terms of previously differentiated mtDNA haplotypes (85,86). However, due to lack of a clear admixture zone, retention of ancestral polymorphisms is the more likely explanation for the pattern in CO1 because in that case, polymorphisms in the common ancestor would arbitrarily be distributed among populations regardless of geography. Thus, CO1 of two sympatric populations could be similar due to incomplete lineage sorting of these ancestral polymorphisms (78).

Population M: The ancestral population along Turkish coasts

Based on this scenario, population *M* would be the local population in the Mediterranean. On the other hand, population *C*, differentiating from population *M* in the Black Sea during the Last Glacial Maximum (LGM) would have subsequently dispersed to eastern Mediterranean after the establishment of the current system in the TSS around 7,000 years ago. The similarity in CO1 data of sympatric populations can be explained by secondary gene flow of mtDNA from the local population *M* to *C*. However, an alternative hypothesis of incomplete lineage sorting in mtDNA is still relevant as two populations would be isolated approximately for only about 15,000 years, which is an inadequate time to detect post-isolation differentiation, considering the slow mutation rate of the CO1 gene (87).

After the opening of the Dardanelles for the first time since the formation of the Tethys Sea in the Riss-Wurm Interglacial (150,000-100,000 ya), ancestral *P. marmoratus* populations might have dispersed from the Mediterranean into the Black Sea, which had higher salinity than the present (20). During the peak of the Last Würm Glaciation (24,000-20,000 ya), the Black Sea population of the species could have survived in isolation, and differentiated from the Mediterranean populations. Under this isolation, population *C* and population *M* would have differentiated in the Black Sea and in the Mediterranean, respectively. Similar suggestions

were also made for the mussel species *P. elegans (37)*, and shrimp species *Crangon crangon (88)* and *P. elegans (89)*.

Afterwards, population *C* would have expanded its range into the Mediterranean when the connection between the Black Sea and the Mediterranean was re-established. In other words, a refugial population of the ancestral *C* population might have survived in the SoM, which probably had relatively higher salinities during the LGM. During its expansion, its mtDNA would have been replaced by that of the local population *M* resulting in lack of differentiation in the mtDNA of sympatric populations. Whereas according to the first scenario, mtDNA of population *C* would be replaced by that of population *M*, here, the opposite is suggested because the assumed local populations are different.

Both mtDNA and nuclear microsatellites showed strong differentiation between populations from the Black Sea and the Mediterranean, complementing the results of Fratini *et al.* (27). This pattern is similar to other species such as anchovies (9), the black scorpionfish *(Scorpaena porcus)* (23), and the Mediterranean mussel *(Mytilus galloprovincialis)* (10), among others. For example, Paterno *et al.* (10), by using genome-wide single nucleotide polymorphisms (SNP), revealed differentiation between the Black Sea and the Mediterranean samples. They also found that Mediterranean populations of *M. galloprovincialis* had mixed ancestry whereas Black Sea populations were more homogeneous. This pattern is similar to more heterogeneous assignment probabilities of *P. marmoratus* individuals in the Mediterranean in our study (Figure 2). Boissin *et al.(23)* also revealed Black Sea-Mediterranean separation in *S. porcus* by microsatellites but not by mtDNA. However, neither of these studies did reveal the pattern around the TSS due to a lack of samples from adjacent basins to TSS (9,23,90).

Biogeographical transition zones present good opportunities for studying the effect of the past ice ages on genetic structure of terrestrial and marine species because secondary contact zones of post-glacial lineages can be created (3,4). In our study, by focusing on one of the less studied transition zones in the Mediterranean, the Turkish Straits System, we found divergent *P. marmoratus* clusters in sympatry in the Eastern Mediterranean basins, suggesting a secondary contact zone between previously isolated populations. This pattern was not detected by mtDNA which mostly reflected the geographic differentiation between the Black Sea and the Mediterranean. Two alternative explanations for cytonuclear pattern in sympatric populations, as retention of ancestral polymorphisms or gene flow after secondary contact could not be distinguished. Most probably, both processes have contributed to the pattern in the process, although the former is more likely, considering the slow mutation rate of CO1. In order to retrieve the complex demographic history and to investigate evolutionary processes resulting in sympatric populations in the Aegean Sea and the Levantine basin, mitochondrial markers with faster mutation rates than CO1 and/or SNP data will be useful.

Acknowledgements

We especially thank Mert Elverici, S. Ünsal Karhan, Kübra Karaman for their help in the fieldwork. We also thank Jukka Jokela and anonymous reviewers for their comments on the paper.

References

1. Toews DPL, Brelsford A. The biogeography of mitochondrial and nuclear discordance in animals. Mol Ecol. 2012 Aug;21(16):3907–30.

2. Ferro I, Morrone JJ. Biogeographical transition zones: A search for conceptual synthesis. Biol J Linn Soc Lond. 2014;113(1):1–12.

3. Swenson NG, Howard DJ. Clustering of Contact Zones, Hybrid Zones, and Phylogeographic Breaks in North America. Am Nat. 2005;166(5):581–91.

4. Rougeux C, Bernatchez L, Gagnaire P-A. Modeling the Multiple Facets of Speciation-with-Gene-Flow toward Inferring the Divergence History of Lake Whitefish Species Pairs (Coregonus clupeaformis). Genome Biol Evol. 2017 Aug 1;9(8):2057–74.

5. Patarnello T, Volckaert FAMJ, Castilho R. Pillars of Hercules: is the Atlantic-Mediterranean transition a phylogeographical break? Mol Ecol. 2007 Nov;16(21):4426–44.

6. Pérez-Losada M, Nolte MJ, Crandall KA, Shaw PW. Testing hypotheses of population structuring in the Northeast Atlantic Ocean and Mediterranean Sea using the common cuttlefish Sepia officinalis. Mol Ecol. 2007;16(13):2667–79.

7. Bekkevold D, André C, Dahlgren TG, Clausen LAW, Torstensen E, Mosegaard H, et al. Environmental correlates of population differentiation in Atlantic herring. Evolution. 2005 Dec;59(12):2656–68.

8. Luttikhuizen PC, Drent J, K T C, Van Der VEER HW, Johannesson K. Genetic architecture in a marine hybrid zone: comparing outlier detection and genomic clines analysis in the bivalve Macoma balthica. Vol. 21, Molecular Ecology. 2012. p. 3048–61.

9. Magoulas A, Castilho R, Caetano S, Marcato S, Patarnello T. Mitochondrial DNA reveals a mosaic pattern of phylogeographical structure in Atlantic and Mediterranean populations of anchovy (Engraulis encrasicolus). Mol Phylogenet Evol. 2006;39:734–46.

10. Paterno M, Bat L, Souissi JB, Boscari E. A Genome-Wide Approach to the Phylogeography of the Mussel Mytilus galloprovincialis in the Adriatic and the Black Seas. Frontiers in Marine Science. 2019;6.

11. Tine M, Kuhl H, Gagnaire P-A, Louro B, Desmarais E, Martins RST, et al. European sea bass genome and its variation provide insights into adaptation to euryhalinity and speciation. Nat Commun. 2014;5(May):5770.

12. Zbawicka M, Sa´nko T, Strand J, Wenne R. New SNP markers reveal largely concordant clinal variation across the hybrid zone between Mytilus spp. in the Baltic Sea. Aquat Biol. 2014 Jun 3;21(1):25–36.

13. Hudson J, Johannesson K, McQuaid CD, Rius M. Secondary contacts and genetic admixture shape colonization by an amphiatlantic epibenthic invertebrate. Evol Appl. 2020 Mar;13(3):600–12.

14. Le Moan A, Gaggiotti O, Henriques R, Martinez P. Beyond parallel evolution: when several species colonize the same environmental gradient. Biorxiv. 2019;

15. Johannesson K, Le Moan A, Perini S, André C. A Darwinian Laboratory of Multiple Contact Zones. Trends Ecol Evol. 2020 Nov;35(11):1021–36.

16. Borsa P, Blanquer A, Berrebi P. Genetic structure of the flounders Platichthys flesus and P. stellatus at different geographic scales. Mar Biol. 1997 Aug 1;129(2):233–46.

17. Pascual M, Rives B, Schunter C, MaCpherson E. Impact of life history traits on gene flow: A multispecies systematic review across oceanographic barriers in the Mediterranean Sea. PLoS One. 2017;12(5):1–20.

18. Aksu AE, Hiscott RN, Kaminski MA, Mudie PJ, Gillespie H, Abrajano T, et al. Last glacial–Holocene paleoceanography of the Black Sea and Marmara Sea: stable isotopic, foraminiferal and coccolith evidence. Mar Geol. 2002;190:119–49.

19. Hiscott RN, Aksu AE, Mudie PJ, Marret F, Abrajano T, Kaminski MA, et al. A gradual drowning of the southwestern Black Sea shelf: Evidence for a progressive rather than abrupt Holocene reconnection with the eastern Mediterranean Sea through the Marmara Sea Gateway. Quat Int. 2007;167-168:19–34.

20. Zaitsev Y, Mamaev V. Biological Diversity in the Black Sea: A Study of Change and Decline. New York: United Nations Publications; 1997.

21. Yebra L, Bonnet D, Harris RP, Lindeque PK, Peijnenburg KT. Barriers in the pelagic: population structuring of Calanus helgolandicus and C. euxinus in European waters. Mar Ecol Prog Ser. 2011;428:135–49.

22. Fontaine MC, Snirc A, Frantzis A, Koutrakis E, Oztürk B, Oztürk AA, et al. History of expansion and anthropogenic collapse in a top marine predator of the Black Sea estimated from genetic data. Proc Natl Acad Sci U S A. 2012 Sep 18;109(38):E2569–76.

23. Boissin E, Micu D, Janczyszyn-Le Goff M, Neglia V, Bat L, Todorova V, et al. Contemporary genetic structure and postglacial demographic history of the black scorpionfish, Scorpaena porcus, in the Mediterranean and the Black Seas. Mol Ecol. 2016;25(10):2195–209.

24. Besiktepe ST, Sur HI, Ozsoy E, Abdul Latif M, Oguz T, Unluata U. The circulation and hydrography of the Marmara Sea. Oceanographic Literature Review. 1995;9(42):704.

25. Oguz T, Özsoy E, Latif MA, Sur HI, Ünlüata Ü. Modeling of Hydraulically Controlled Exchange Flow in the Bosphorus Strait. J Phys Oceanogr. 1990 Jul 1;20(7):945–65.

26. Sur HI, Ozsoy E, Ilyin YP, Unluata U. Coastal/deep ocean interactions in the Black Sea and their ecological/environmental impacts. J Mar Syst. 1996;7(2):293–320.

27. Fratini S, Ragionieri L, Deli T, Harrer A, Marino IAM, Cannicci S, et al. Unravelling population genetic structure with mitochondrial DNA in a notional panmictic coastal crab species: sample size makes the difference. BMC Evol Biol. 2016;16(1):1–15.

28. Ivanova PP, Dobrovolov IS. Population–genetic structure on European anchovy (Engraulis encrasicolus, linnaeus, 1758) (osteichthyes: engraulidae) from mediterranean basin and Atlantic ocean. Acta Adriat. 2006;(47):13–22.

29. Turan C, Gurlek M, Yaglioglu D, Ozturk B. Genetic differentiation of Mediterranean horse mackerel (Trachurus mediterraneus) populations as revealed by mtDNA PCR-RFLP analysis. J Appl Ichthyol. 2009;(25):142–7.

30. Rossi AR, Crosetti D, Livi S. Genetics of mugilidae. In: Crosetti D, Blaber S, editors. Biology, Ecology and Culture of Grey Mullet (Mugilidae). CRC Press, Boca Raton, USA; 2015. p. 349–97.

31. Turan C, Gurlek M, Erguden D, Yaglioglu D, Uyan A, Reyhaniye AN, et al. Population Genetic

Analysis of Atlantic bonito Sarda sarda (Bloch, 1793) Using Sequence Analysis of mtDNA D-loop Region. Fresenius Environ Bull. 2015;(24):3148–54.

32. Turan C. Microsatellite DNA reveals genetically different populations of Atlantic bonito Sarda sarda in the Mediterranean Basin. Biochem Syst Ecol. 2015;63:174–82.

33. Viaud-Martínez KA, Martínez Vergara M, Goldin PE, Ridoux V, Öztürk AA, Öztürk B, et al. Morphological and genetic differentiation of the Black Sea harbour porpoise Phocoena phocoena. Mar Ecol Prog Ser. 2007;338:281–94.

34. Tonay AM, Yazıcı Ö, Dede A, Bilgin S, Danyer E, Aytemiz I, et al. Is there a distinct harbor porpoise subpopulation in the Marmara Sea? Mitochondrial DNA Part A: DNA Mapping, Sequencing, and Analysis. 2016;28:558–64.

35. Kalkan E, Karhan SU, Yokes MB, Bilgin R. The Turkish Straits System–phylogeographic break for the caridean shrimp Palaemon elegans (Crustacea: Decapoda). Rapp Comm Int Mer Médit. 2013;40:775.

36. Kalkan E, Bilgin R. The intertwined effects of geographic barriers, palaeoclimate and life history on the distribution of genetic diversity: A case study with two marine species in the Eastern Mediterranean. In: 3rd Ecology and Evolutionary Biology Symposium. 2016.

37. Kalkan E, Karhan SU, Bilgin R. Population genetic structure of the marbled crab, Pachygrapsus marmoratus from Turkish coasts of the Black Sea and the Eastern Mediterranean. In: Rapports et procès verbaux des réunions, Commission internationale pour l'exploration scientifique de la Mer Méditerranée. 2013. p. 713.

38. Deli T, Pfaller M, Schubart C. Phylogeography of the littoral prawn species Palaemon elegans (Crustacea: Caridea: Palaemonidae) across the Mediterranean Sea unveils disparate patterns of population genetic structure and demographic history in the two sympatric genetic types II and III. Mar Biodivers. 2017;48(4):1979–2001.

39. Warburg MR, Goldenberg S, Tudiver B. Osmotic and ionic regulation in two Pachygrapsus crabs, under varying salinities and dehydration. Comp Biochem Physiol A Physiol. 1987;86(4):761–5.

40. Madeira D, Narciso L, Cabral HN, Vinagre C. Thermal tolerance and potential impacts of climate change on coastal and estuarine organisms. J Sea Res. 2012;70:32–41.

41. Flores A a. V, Paula J. Population dynamics of the shore crab Pachygrapsus marmoratus (Brachyura: Grapsidae) in the central Portuguese coast. Journal of the Marine Biological Association of the UK. 2002;82:229–41.

42. Cuesta JA, Rodríguez A. Zoeal stages of the intertidal crab Pachygrapsus marmoratus (Fabricius, 1787) (Brachyura, Grapsidae) reared in the laboratory. Hydrobiologia. 2000;436:119–30.

43. Fratini S, Zane L, Ragionieri L, Vannini M, Cannicci S. Relationship between heavy metal accumulation and genetic variability decrease in the intertidal crab Pachygrapsus marmoratus (Decapoda; Grapsidae). Estuar Coast Shelf Sci. 2008;79:679–86.

44. Fratini S, Schubart C, Ragionieri L. Population genetics in the rocky shore crab Pachygrapsus marmoratus from the western Mediterranean and eastern Atlantic : complementary results from mtDNA and microsatellites at different geographic scales. Crustac Issues. 2011;(June 2016):191–9.

45. Fratini S, Ragionieri L, Cutuli G, Vannini M, Cannicci S. Pattern of genetic isolation in the crab Pachygrapsus marmoratus within the Tuscan Archipelago (Mediterranean Sea). Mar Ecol Prog

Ser. 2013 Mar 25;478:173–83.

46. Deli T, Bahles H, Said K, Chatti N. Patterns of genetic and morphometric diversity in the marbled crab (Pachygrapsus marmoratus, Brachyura, Grapsidae) populations across the Tunisian coast. Acta Oceanol Sin. 2015 Jun 1;34(6):49–58.

47. Deli T, Fratini S, Ragionieri L, Said K, Chatti N, Schubart CD. Phylogeography of the marbled crab Pachygrapsus marmoratus (Decapoda, Grapsidae) along part of the African Mediterranean coast reveals genetic homogeneity across the Siculo-Tunisian Strait versus heterogeneity across the Gibraltar Strait. Mar Biol Res. 2016;12(5):471–87.

48. Avise JC. Molecular Markers, Natural History and Evolution: Natural History and Evolution. Springer Science & Business Media; 1994. 511 p.

49. Silva IC, Mesquita N, Schubart CD, Alves MJ, Paula J. Genetic patchiness of the shore crab Pachygrapsus marmoratus along the Portuguese coast. J Exp Mar Bio Ecol. 2009;378(1-2):50–7.

50. Iannucci A, Cannicci S, Caliani I, Baratti M, Pretti C, Fratini S. Investigation of mechanisms underlying chaotic genetic patchiness in the intertidal marbled crab Pachygrapsus marmoratus (Brachyura: Grapsidae) across the Ligurian Sea. BMC Evol Biol. 2020 Aug 24;20(1):108.

51. Fratini S, Ragionieri L, Papetti C, Pitruzzella G, Rorandelli R, Barbaresi S, et al. Isolation and characterization of microsatellites in Pachygrapsus marmoratus (Grapsidae; Decapoda; Brachyura). Mol Ecol Notes. 2006;6:179–81.

52. Amos W, Hoffman JI, Frodsham A, Zhang L, Best S, Hill AVS. Automated binning of microsatellite alleles: Problems and solutions. Mol Ecol Notes. 2007;7:10–4.

53. Van Oosterhout C, Hutchinson WF, Wills DPM, Shipley P. MICRO-CHECKER: Software for identifying and correcting genotyping errors in microsatellite data. Mol Ecol Notes. 2004;4:535–8.

54. Brookfield JFY. A simple new method for estimating null allele frequency from heterozygote deficiency. Mol Ecol. 1996;5(3):453–5.

55. Pritchard JK, Stephens M, Donnelly P. Inference of population structure using multilocus genotype data. Genetics. 2000;155:945–59.

56. Evanno G, Regnaut S, Goudet J. Detecting the number of clusters of individuals using the software STRUCTURE: A simulation study. Mol Ecol. 2005;14:2611–20.

57. Earl DA, vonHoldt BM. STRUCTURE HARVESTER: a website and program for visualizing STRUCTURE output and implementing the Evanno method. Conserv Genet Resour. 2012 Jun;4(2):359–61.

58. Kopelman NM, Mayzel J, Jakobsson M, Rosenberg NA, Mayrose I. Clumpak: a program for identifying clustering modes and packaging population structure inferences across K. Mol Ecol Resour. 2015 Sep;15(5):1179–91.

59. Jakobsson M, Rosenberg NA. CLUMPP: A cluster matching and permutation program for dealing with label switching and multimodality in analysis of population structure. Bioinformatics. 2007;23(14):1801–6.

60. Rosenberg NA. distruct: a program for the graphical display of population structure. Mol Ecol Notes. 2003 Dec 10;4(1):137–8.

61. Raymond M, Rousset F. Genepop (Version-1.2) - Population-Genetics Software for Exact Tests

and Ecumenicism. J Hered. 1995;86:248–9.

62. Rousset F. GENEPOP'007: A complete re-implementation of the GENEPOP software for Windows and Linux. Mol Ecol Resour. 2008;8:103–6.

63. Excoffier L, Smouse PE, Quattro JM. Analysis of molecular variance inferred from metric distances among DNA haplotypes – application to human mitochondrial-DNA restriction data. Genetics. 1992;131:479–91.

64. Excoffier L, Lischer HEL. Arlequin suite ver 3.5: A new series of programs to perform population genetics analyses under Linux and Windows. Mol Ecol Resour. 2010;10:564–7.

65. Rice WR. Analyzing Tables of Statistical Tests. Evolution. 1989;43:223–5.

66. Peakall R, Smouse P. Spatial autocorrelation analysis of individual multiallele and multilocus genetic structure. Heredity . 1999;(82):561–73.

67. Peakall R, Smouse PE. GENALEX 6: Genetic analysis in Excel. Population genetic software for teaching and research. Mol Ecol Notes. 2006;6:288–95.

68. Peakall R, Smouse PE. GenALEx 6.5: Genetic analysis in Excel. Population genetic software for teaching and research-an update. Bioinformatics. 2012;28(19):2537–9.

69. Folmer OM Black R Hoeh R Lutz R VR. DNA primers for amplification of mitochondrial cytochrome c oxi- dase subunit I from diverse metazoan invertebrates. Mol Mar Biol Biotechnol. 1994;(3):294–9.

70. Thompson JD, Gibson TJ, Plewniak F, Jeanmougin F, Higgins DG. The CLUSTAL X windows interface: Flexible strategies for multiple sequence alignment aided by quality analysis tools. Nucleic Acids Res. 1997;25(24):4876–82.

71. Bandelt HJ, Forster P, Röhl A. Median-joining networks for inferring intraspecific phylogenies. Mol Biol Evol. 1999 Jan;16(1):37–48.

72. Librado P, Rozas J. DnaSP v5: A software for comprehensive analysis of DNA polymorphism data. Bioinformatics. 2009;25(11):1451–2.

73. Tajima F. Statistical method for testing the neutral mutation hypothesis by DNA polymorphism. Genetics. 1989;123:585–95.

74. Fu YX, Li WH. Statistical tests of neutrality of mutations. Genetics. 1993 Mar;133(3):693–709.

75. Fu YX. Statistical tests of neutrality of mutations against population growth, hitchhiking and background selection. Genetics. 1997 Oct;147(2):915–25.

76. Ramos-Onsins SE, Rozas J. Statistical Properties of New Neutrality Tests Against Population Growth. Mol Biol Evol. 2006 Aug 1;23(8):1642–1642.

77. Ramírez-Soriano A, Ramos-Onsins SE, Rozas J, Calafell F, Navarro A. Statistical power analysis of neutrality tests under demographic expansions, contractions and bottlenecks with recombination. Genetics. 2008 May;179(1):555–67.

78. Funk DJ, Omland KE. Annual Review of Ecology, Evolution, and Systematics,. Annu Rev Ecol Syst. 2003;34:397–423.

79. Ünlülata Ü, Oğuz T, Latif MA, Özsoy E. On the Physical Oceanography of the Turkish Straits. In: Pratt LJ, editor. The Physical Oceanography of Sea Straits. Dordrecht: Springer Netherlands;

1990. p. 25–60.

80. Jarosz E, Teague WJ, Book JW, Beşiktepe ŞT. Observations on the characteristics of the exchange flow in the Dardanelles Strait. J Geophys Res. 2012 Nov;117(C11).

81. Sannino G, Sözer A, Özsoy E. A high-resolution modelling study of the Turkish Straits System. Ocean Dyn. 2017 Apr 1;67(3):397–432.

82. Ünlüata U, Oğuz T, Latif MA, Özsoy E, Pratt LJ. The physical oceanography of sea straits. 1990;

83. Peijnenburg KTCA, Fauvelot C, Breeuwer JAJ, Menken SBJ. Spatial and temporal genetic structure of the planktonic Sagitta setosa (Chaetognatha) in European seas as revealed by mitochondrial and nuclear DNA markers. Mol Ecol. 2006;15:3319–38.

84. Svitoch AA, Selivanov AO, Yanina TA. Paleohydrology of the Black Sea Pleistocene basins. Water Resour. 2000;27(6):594–603.

85. Currat M, Ruedi M, Petit RJ, Excoffier L. The hidden side of invasions: Massive introgression by local genes. Evolution. 2008;62(8):1908–20.

86. Excoffier L, Foll M, Petit RJ. Genetic Consequences of Range Expansions. Annu Rev Ecol Evol Syst. 2009;40(1):481–501.

87. Schubart CD, Diesel R, Hedges SB. Rapid evolution to terrestrial life in Jamaican crabs. Nature. 1998 May 28;393(6683):363–5.

88. Luttikhuizen PC, Campos J, van Bleijswijk J, Peijnenburg KTCA, van der Veer HW. Phylogeography of the common shrimp, Crangon crangon (L.) across its distribution range. Mol Phylogenet Evol. 2008;46:1015–30.

89. Reuschel S, Cuesta JA, Schubart CD. Marine biogeographic boundaries and human introduction along the European coast revealed by phylogeography of the prawn Palaemon elegans. Mol Phylogenet Evol. 2010 Jun;55(3):765–75.

90. Durand JD, Blel H, Shen KN, Koutrakis ET, Guinand B. Population genetic structure of Mugil cephalus in the Mediterranean and Black Seas: a single mitochondrial clade and many nuclear barriers. Mar Ecol Prog Ser. 2013 Jan 31;474:243–61.

Supporting information

S1 Table. Sampling sites and their corresponding coordinates. Codes indicate the abbreviations used in the map in Fig 1.

S2 Table. Percentages of loci with significant null alleles in 20 populations. Significant loci determined by HWE test after Bonferroni correction are in bold.

S3 Table. Average *Fst* for all loci and individual contribution of loci for *Fst* values between populations. *Fst* values calculated with FreeNA corrected genotypes and not-corrected genotypes are indicated.

Click here to access/download;Figure;Fig1.tif

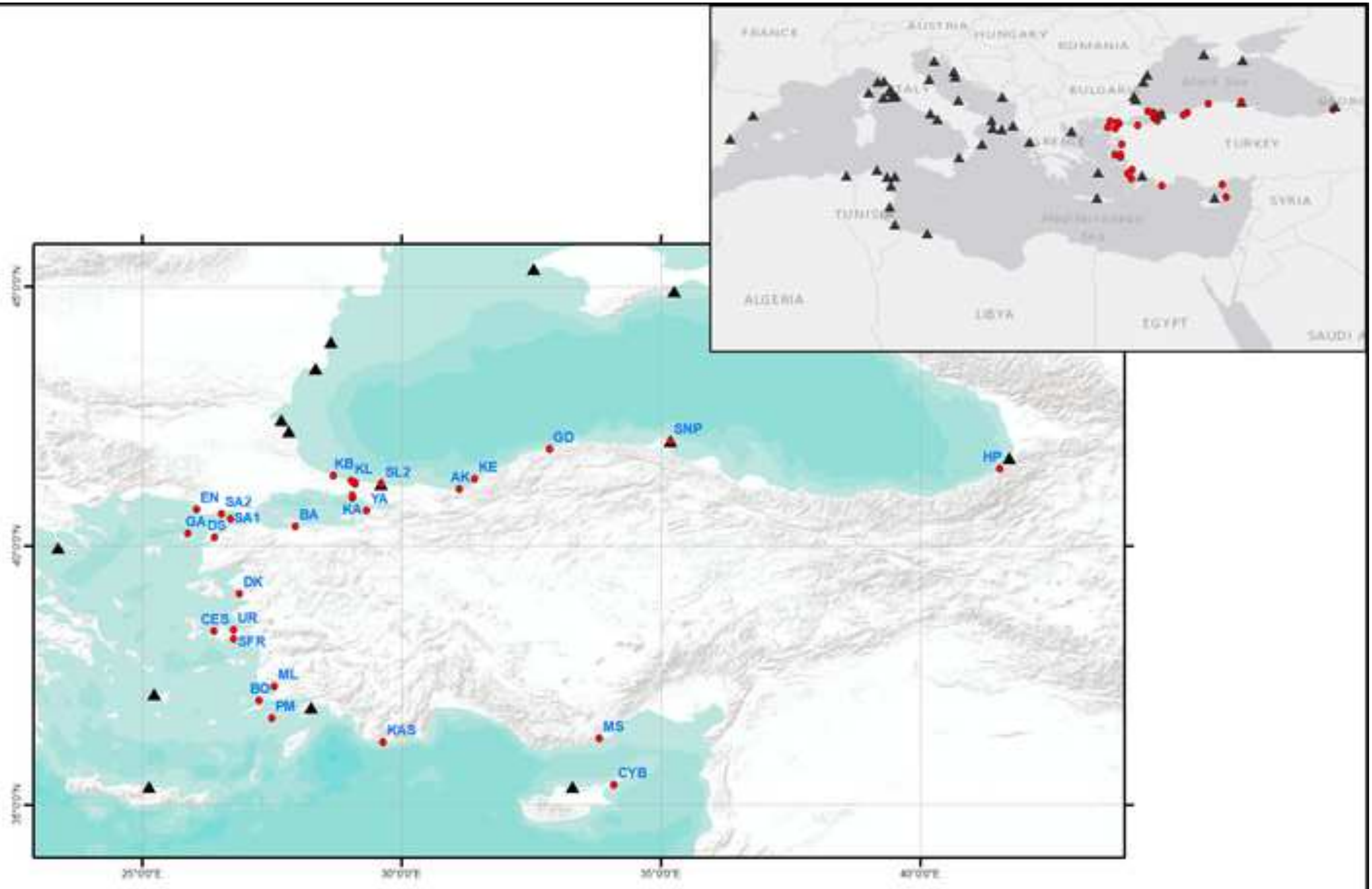

Service Layer Credits: Sources: Esri, USGS, NOAA
Esri, HERE, DeLorme, MapmyIndia, © OpenStreetMap contributors, and the GIS user community

Click here to access/download;Figure;Fig2.tif

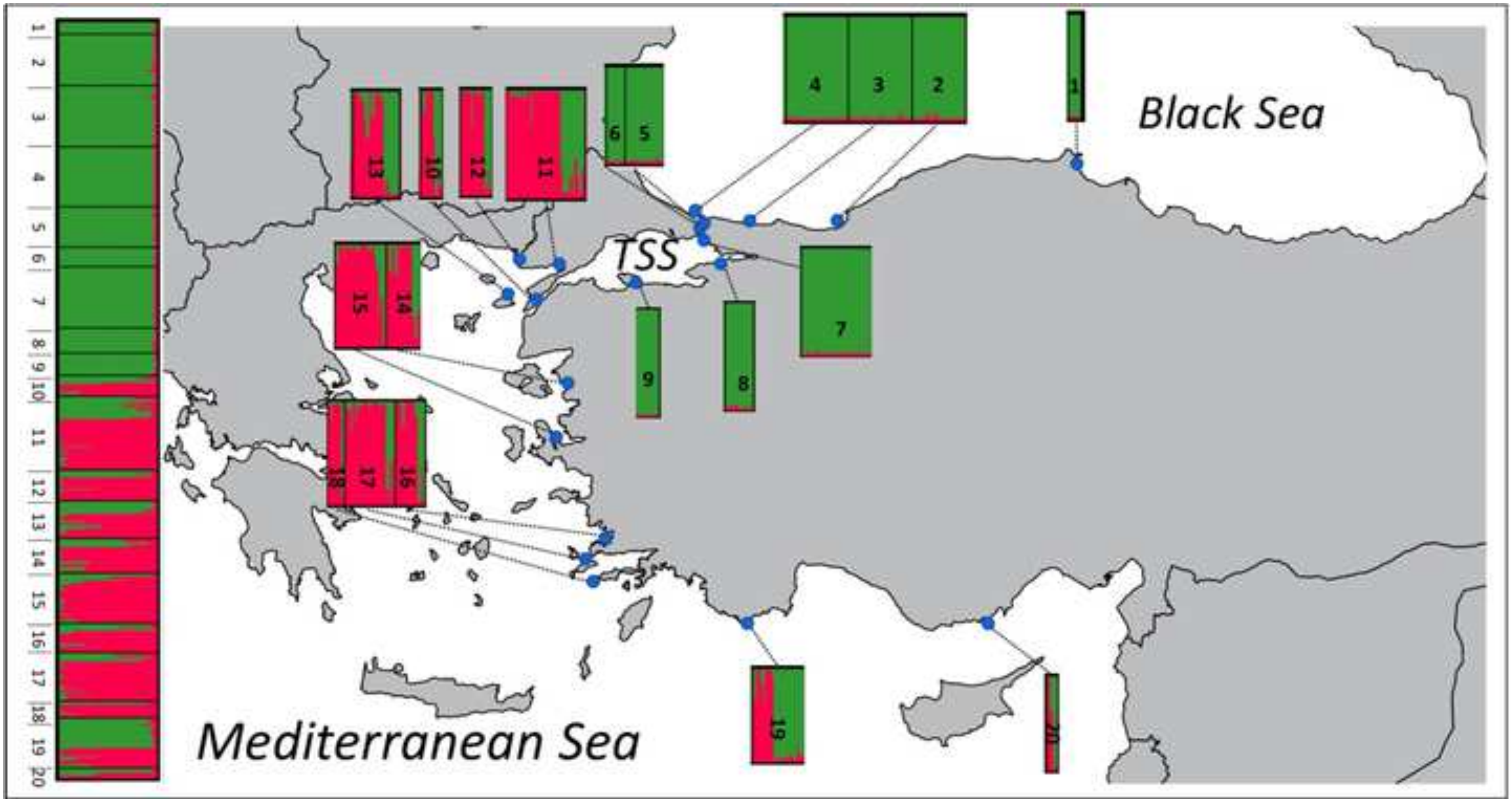

Click here to access/download;Figure;Fig3.tif

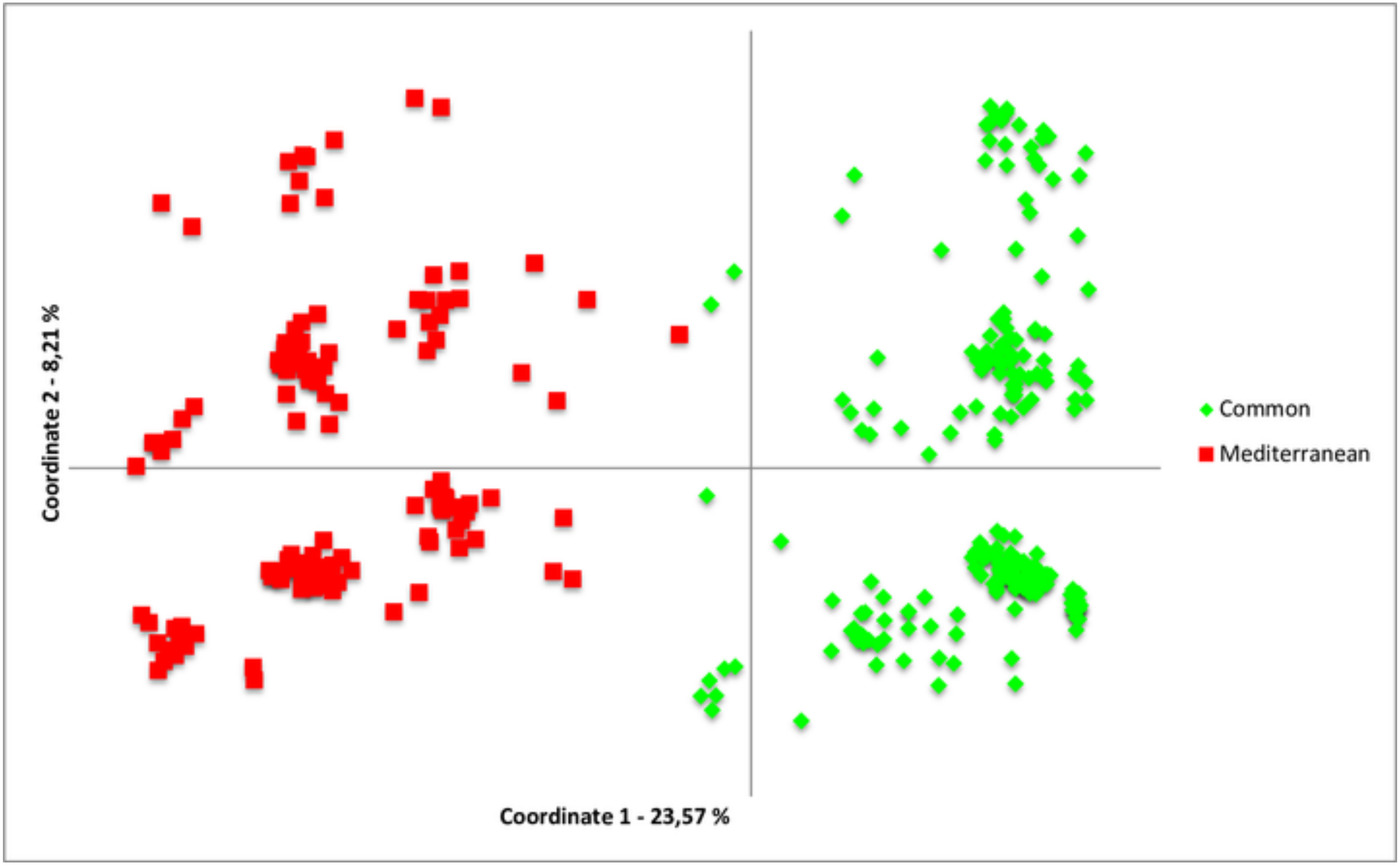

Click here to access/download;Figure;Fig4.tif

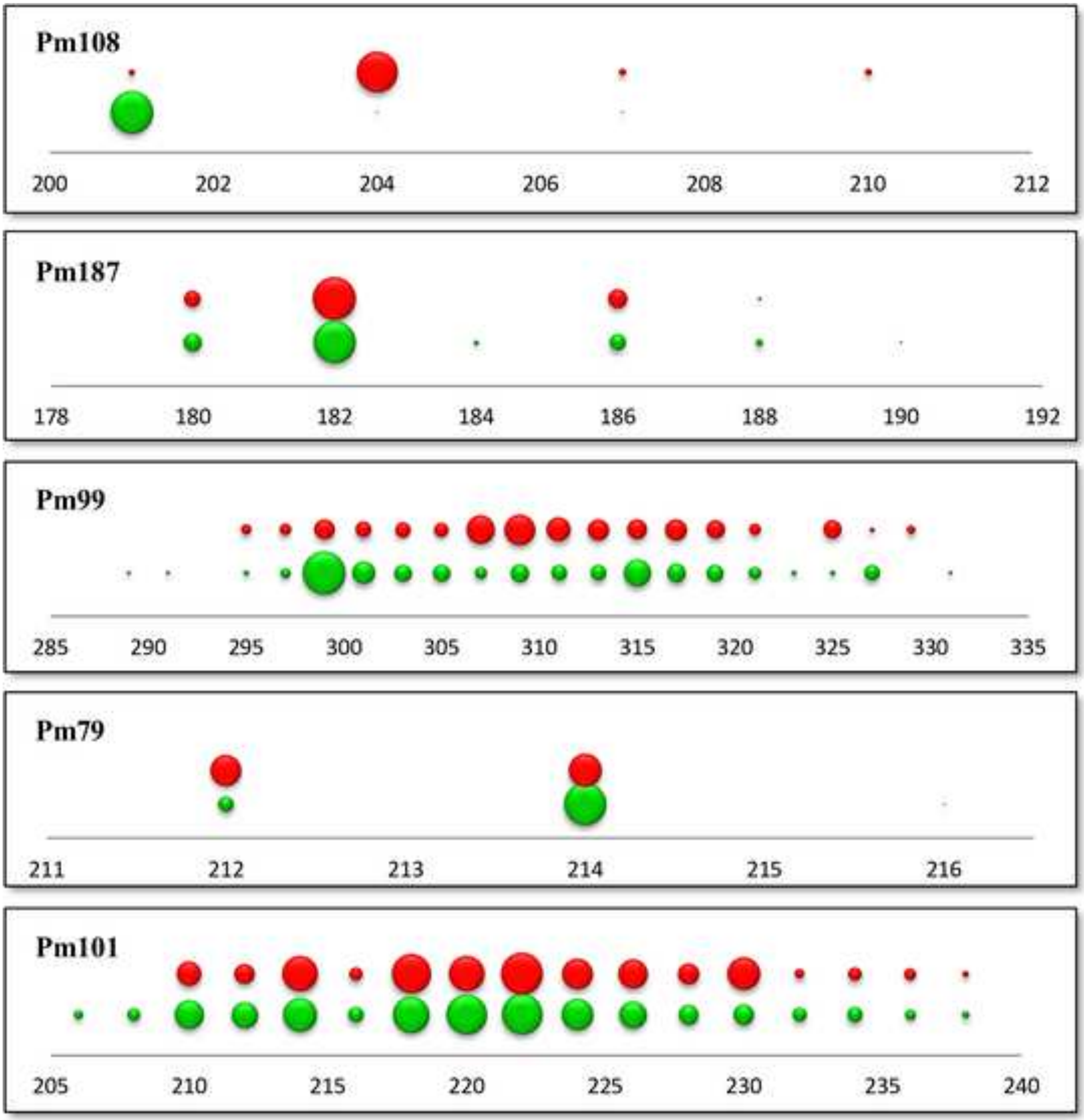

Click here to access/download;Figure;Fig5.tif

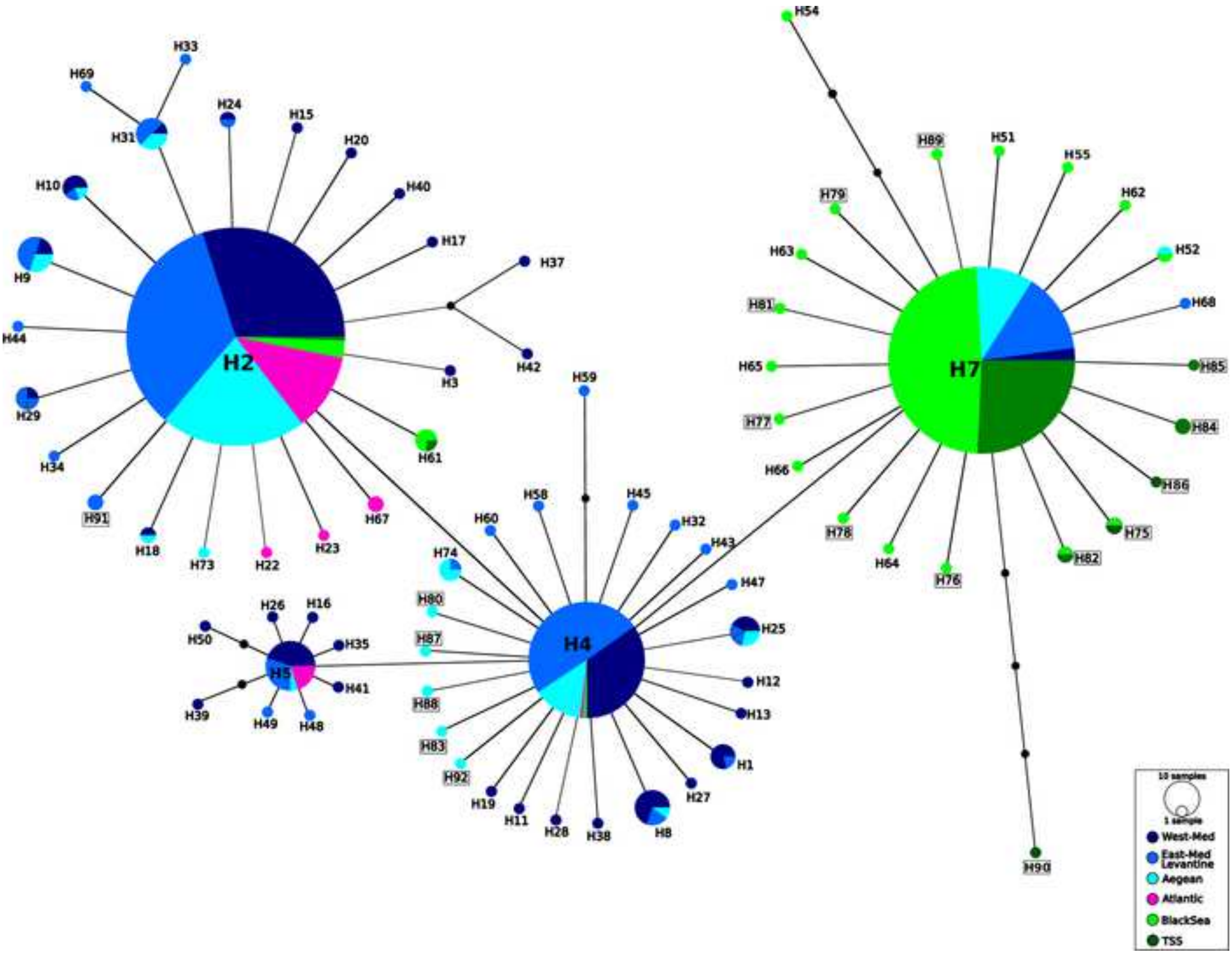

Click here to access/download
Supporting Information
S1_Fig.pdf

Click here to access/download
Supporting Information
S1_Table.pdf

Click here to access/download
Supporting Information
S2_Table.pdf

Click here to access/download
Supporting Information
S3_Table.pdf
