# Supplementary material for: Mitonuclear genetic patterns of divergence in the marbled crab, Pachygrapsus marmoratus (Fabricius, 1787) along the Turkish seas
Source: PLoS One. 2022 Apr 5;17(4):e0266506. doi: 10.1371/journal.pone.0266506 (PMC8982882; doi:10.1371/journal.pone.0266506)
Supplement: Attachment — Submitted filename: PlosOne_Reviewers.docx [file pone.0266506.s014.docx]

PLOS ONE

Additional Editor Comments:

The manuscript is greatly improved and the reviewers were generally pleased with the ammendments. Few minor things remain.

*We thank the editor and the reviewers for their comments and for the opportunity to submit a revised draft. Below, we answer the specific comments and refer to changes we made.*

Main points.
1. The higher sign in AMOVA with clusters derived from the data is ENTIRELY expected. the data is analyzed for structure, and then the data are analyzed with respect to that structure (circlular). I recommend you drop the Amova on clusters, and only keep the geographic regions and Sampling site anova up in the table. You can test in cross, variance in microsats within clusters defiend from mtDNA – and vice versa.

*Yes, we agree that it is expected to have the same signal in AMOVA as STRUCTURE clusters for microsatellites. Thus, we removed that part from Table 1. However, we kept the part where we tested variance in mtDNA explained by microsatellite clusters in Table 3, as it is a cross-test.*

2. Rev 2 asked for Samova analyses. I say more analyses are not needed (unless you want to give it a go). But you should discuss that doing Samova might be interesting (also on more markers) and the K3-5 heterogeneity shown by the structure analyses, and that possible explanations for these results. Are these non-biological (technical), multiple ancestral populations that mixed, or ecomorphs within species,…etc

*Thank you very much for your comment. We agree that new analyses are not needed. Since the K3-5 heterogeneity of the STRUCTURE analysis mainly involves cluster C with no geographical pattern (they are both present in the Black Sea and the Mediterranean), we believe this could be an artefact of the small number of markers used or a reflection of chaotic genetic patchiness. We also think further analyses with more markers will be needed and interesting to understand the detailed geographical pattern better.*

*Now we also added a paragraph in our discussion about this:*

*Line 521-532: “Larger genetic variation within sampling sites than those between them (see Table 1 and S5 Fig) suggest a heterogenic genetic pool which was also observed in previous studies across the Italian [47–49], Tunisian [51], Portuguese [53] and Ligurian coasts [54]. Additionally, K=3 barplot of the STRUCTURE analysis showed heterogeneity of sampling sites mainly involving cluster C with no geographical structure (S2 Fig). Previously suggested ecological and biological processes such as differences in reproductive success, larval dispersion pattern and local larval retention could also be responsible for heterogeneity of the genetic pool observed in this study [54]. However, heterogeneity of STRUCTURE clusters at K 3-5 could also be an artefact of the small number of markers used. We think that by using more genetic markers such as SNPs, better inferences on the drivers of genetic structure in the region could be possible with more detailed analyses such as Spatial Analysis of Molecular Variance (SAMOVA) and genetic relatedness.”*

3. Check that all figures and tables including for the supplementary is refereed correctly as e.g., the S2 figure is not refereed to in the text. At the same time S1 Fig, S2 Table, and S3 Table was not included in the submission (rev 2).

*Thank you, now all of the supplementary material is included. S2 Figure is also referred to in the text:*

*Line 297: “Barplots for other K values (K: 3-5) were visually inspected (S2 Fig). At K=3, some individuals belonging to Cluster C formed a third cluster, but this did not correspond to any geographical pattern (S2 Fig).”*

Minor points.
New opening line of abstract is better, but the ending is a bit strange. Consider rewriting and switching verbs, “created” seems a bit off.
“because secondary contact zones of post-glacial lineages can be created.”

*Thank you, we now changed the verb to “formed” (Line 35).*

Line 84. Add reference
“These transition zones are often regions where divergent lineages meet in secondary contact.”

*A reference is now added here accordingly (Line 71).*

6. Review Comments to the Author

Please use the space provided to explain your answers to the questions above. You may also include additional comments for the author, including concerns about dual publication, research ethics, or publication ethics. (Please upload your review as an attachment if it exceeds 20,000 characters)

Reviewer #1: I’m happy with the amendments the authors have made and want to congratulate the authors on making it this far. As I’m happy with the amendments, I don’t have any more comments, except that the authors need to check that all figures and tables including for the supplementary is refereed correctly as e.g., the S2 figure is not refereed to in the text. At the same time S1 Fig, S2 Table, and S3 Table was not included in the submission. But even so I’m happy with the amendments.

Reviewer #2: General

I think the manuscript has been considerably improved and my major concerns have been mostly resolved. However, I still have one concern about the results of AMOVA.

Major comments:

Regarding comments by Reviewer 1 (32) and reply to my previous comment (45): I agree with Reviewer 1. The AMOVA results should be more carefully discussed. I think the AMOVA test showing more variation within groups than between groups implies that the grouping by geographical regions does not sufficiently represent population genetic structure.
I therefore recommend you to conduct SAMOVA with sampling site data, not by regions or clusters C/M, since SAMOVA can detect the most plausible grouping by comparing FCT values of different K without information for grouping in advance. I do not agree with your opinion that "SAMOVA won't add anything new to our interpretation."

*Thank you very much for your suggestion. In addition to our previous answer to Reviewer 1 (32), we think Fst table and Structure map would show the signal if SAMOVA would be picking up a previously undetected pattern. Additionally, PcoA analysis (S5 Fig) does not show any groupings even within the sampling sites. However, we believe that with more genetic markers (such as SNPs) and sampling effort, more detailed analyses such as SAMOVA could be useful for inferring the drivers of genetic structure in the region.*

*We now also discuss AMOVA in this perspective in the discussion:*

*Line 521-532: “Larger genetic variation within sampling sites than those between them (see Table 1 and S5 Fig) suggest a heterogenic genetic pool which was also observed in previous studies across the Italian [47–49], Tunisian [51], Portuguese [53] and Ligurian coasts [54]. Additionally, K=3 barplot of the STRUCTURE analysis showed heterogeneity of sampling sites mainly involving cluster C with no geographical structure (S2 Fig). Previously suggested ecological and biological processes such as differences in reproductive success, larval dispersion pattern and local larval retention could also be responsible for heterogeneity of genetic pool in this study [54]. However, heterogeneity of STRUCTURE clusters at K 3-5 could also be an artefact of the small number of markers used. We think that using more genetic markers such as SNPs, better inferences of the drivers of genetic structure in the region could be possible with more detailed analyses such as Spatial Analysis of Molecular Variance (SAMOVA) and genetic relatedness”*

Minor comments:

Line 44. SoM is not obvious in Abstract. Please spell out it for the first appearance.

*Thank you, now it is spelled out (Line 44).*

Line 126. Mediterranean => the Mediterranean / Gbif => GBIF

*Corrected accordingly.(Line 130)*

Line 142. ", [27]" => " [27], "

*Corrected accordingly (now it is the reference [30] instead of 27.). (Line 146)*

Line 280–282. I think it is also important for Pm79 that numbers of 212 and 214 are almost same in red but 214 dominates in green.

We now added a sentence to the next paragraph about this:
*Line 310: “…Cluster C had a dominant allele for the locus Pm79 whereas M had almost equal frequencies of its alleles.”.*

Line 494-496. Explaining the similarity of COI sequences by its slow mutation rate may contradict your discussion in Line 438-440 that mtDNA sorts faster.

*Here, mtDNA sorting faster because of its smaller effective population size does not necessarily contradict our discussion. Because of this, we see a stronger effect of genetic drift in the Black Sea in the mtDNA. On the other hand, mutation rate of COI would be too slow for new haplotypes appearing and becoming prevalent in the last 20,000 years. As a result, it does not have enough resolution to help infer processes during the LGM, and thus could explain the similarity of haplotypes in the Mediterranean Sea.*

Reviewer #3: The authors have incorporated the comments and suggestions to the manuscript, with the exception of one suggested analysis (IMA), which they have provided justification for (results do not provide meaningful estimates of divergence time because of very wide variation). The manuscript is much improved incorporating all the other comments from the reviewers.

*Thank you very much.*
